# Synthesis, anti-bacterial and anti-protozoal activities of amidinobenzimidazole derivatives and their interactions with DNA and RNA

**DOI:** 10.1080/14756366.2018.1484733

**Published:** 2018-08-31

**Authors:** Andrea Bistrović, Luka Krstulović, Ivana Stolić, Domagoj Drenjančević, Jasminka Talapko, Martin C. Taylor, John M. Kelly, Miroslav Bajić, Silvana Raić-Malić

**Affiliations:** a Department of Organic Chemistry, Faculty of Chemical Engineering and Technology, University of Zagreb, Zagreb, Croatia;; b Department of Chemistry and Biochemistry, Faculty of Veterinary Medicine, University of Zagreb, Zagreb, Croatia;; c Department of Transfusion Medicine, Osijek University Hospital, Osijek, Croatia;; d Department of Microbiology and Parasitology, Faculty of Medicine, University of Osijek, Osijek, Croatia;; e Department of Pathogen Molecular Biology, London School of Hygiene and Tropical Medicine, London, UK

**Keywords:** Benzimidazole, 1,2,3-triazole, resistant bacteria, antiprotozoal activity, *Trypanosoma brucei*, MRSA

## Abstract

Amidinobenzimidazole derivatives connected to 1-aryl-substituted 1,2,3-triazole through phenoxymethylene linkers **7a**–**7e**, **8a**–**8e**, and **9a**–**9e** were designed and synthesised with the aim of evaluating their anti-bacterial and anti-trypanosomal activities and DNA/RNA binding affinity. Results from anti-bacterial evaluations of antibiotic-resistant pathogenic bacteria revealed that both *o*-chlorophenyl-1,2,3-triazole and *N*-isopropylamidine moieties in **8c** led to strong inhibitory activity against resistant Gram-positive bacteria, particularly the MRSA strain. Furthermore, the non-substituted amidine and phenyl ring in **7a** induced a marked anti-bacterial effect, with potency against ESBL-producing Gram-negative *E. coli* better than those of the antibiotics ceftazidime and ciprofloxacin. UV–Vis and CD spectroscopy, as well as thermal denaturation assays, indicated that compounds **7a** and **8c** showed also binding affinities towards *ct*DNA. Anti-trypanosomal evaluations showed that the *p*-methoxyphenyl-1,2,3-triazole moiety in **7b** and **9b** enhanced inhibitory activity against *T. brucei*, with **8b** being more potent than nifurtimox, and having minimal toxicity towards mammalian cells.

## Graphical Abstract

**Figure:**
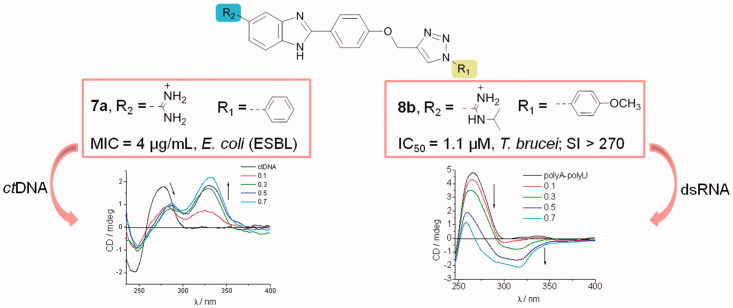

## Introduction

The benzimidazole derivatives, which contain fused heterocyclic nuclei within their structures, are structural isosteres of purine bases. This allows them to interact with biopolymers and they, therefore, have diverse biological and clinical applications^1–5^. Much research effort has been aimed at targeting DNA with benzimidazole ligands, with the goal of designing agents that have therapeutic applications^5–9^. Although RNA is a well-established target of current antibiotics, designing new compounds that selectively recognise RNA has also been a difficult task, particularly when focused on the treatment of a variety of infections^10–12^. The challenge is to produce drug-like molecules with high affinity for DNA/RNA, while maintaining sufficient sequence selectivity. While there are many areas of therapy that might benefit from DNA-directed intervention, there is currently an urgent need for new antimicrobials with novel modes of action.

Antibiotic resistance is a global public threat because of its effect on health care with prolonged hospitalisations and increased mortality. The increasing prevalence of hospital and community-acquired infections caused by multidrug-resistant (MDR) bacterial pathogens is limiting the options for effective antibiotic therapy^13^ ^,^ ^14^. Drug-resistant Gram-positive bacterial pathogens, including methicillin-resistant *Staphylococcus aureus* (MRSA) and vancomycin-resistant *enterococci* (VRE), have become a serious clinical problem that impinges on the treatment of various nosocomial and community-acquired infections^15^ ^,^ ^16^. In addition, an increased incidence of MDR Gram-negative bacteria, such as *Pseudomonas aeruginosa*, *Escherichia coli*, and *Klebsiella pneumoniae*, coupled with the lack of novel antibiotics, represents one of the biggest threats to the control of respiratory and other infections^17^. In order to overcome these emerging bacterial resistance problems, novel anti-bacterial drugs need to be developed. Accordingly, in recent years, numerous efforts have focused on discovering novel benzimidazole-based anti-bacterial agents^18–24^. The importance of a protonable chemical moiety within anti-bacterial drugs has been investigated in different studies^25^ ^,^ ^26^. These have revealed the significant uptake of amidine-containing DNA ligands into bacteria, and also into the nuclei of eukaryotic cells^27^. In addition, the structural features of 1,2,3-triazole also enable it to mimic different functional groups, justifying its wide use as a bioisostere for the design of antimicrobial drug analogs^28^ ^,^ ^29^. For example 1,4-disubstituted 1,2,3-triazoles are good *Z*-amide isosteres, because the C-4 atom can act as an electrophilic site; the CH bond (in the 5-position) acts as a hydrogen bond donor, and the lone pair of N-3 electrons acts as a hydrogen bond acceptor^30^.

**Figure 1.**
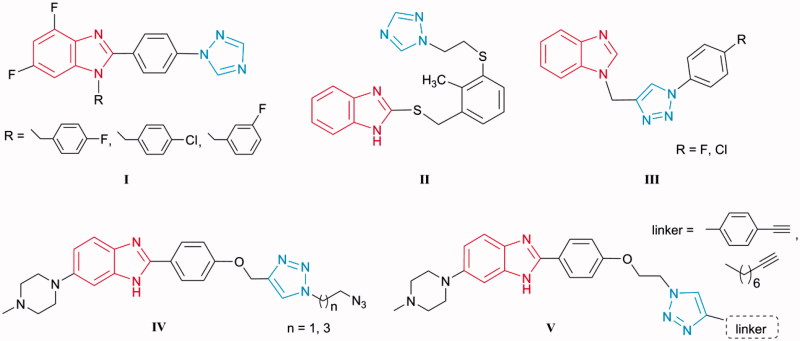
Representatives of benzimidazoles containing triazole moiety I–V as potential antibacterial agents.

A wide range of pharmacological activities has been attributed to the unusual chemical features of azole rings, such as benzimidazole and 1,2,3-triazole. These are able to interact in a non-covalent way with a range of targets, due to the presence of an electron-rich aromatic system and heteroatoms^31^ ^,^ ^32^, and act as promising moieties for the design of novel scaffolds with anti-bacterial activity. Thus, among the series of [1,2,4-triazolyl]phenyl-substituted 4,6-difluorobenzimidazoles **I** ^33^, analogues with electronegative substituents emerged as promising antimicrobials, while 2-thiobenzimidazole with [(1,2,4-triazolyl)ethylthio]phenyl moiety **II** ^34^ exhibited anti-bacterial properties that were selective for *Helicobacter pylori* (Figure 1). Benzimidazole–1,2,3-triazole conjugates **III** with aromatic (*p*-chlorophenyl and *p*-fluorophenyl) 4-substituted triazoles exhibited selective anti-*Moraxella catarrhalis* activity^35^. Furthermore, triazole-bearing monobenzimidazoles **IV** and **V** inhibited growth of Gram-positive bacteria, including two MRSA strains, and displayed *E. coli* DNA topoisomerase I inhibition^36^.

The increasingly important role of benzimidazole and triazole derivatives has been also demonstrated by their *in vivo* evaluations against Gram-positive^37–42^ and Gram-negative bacteria^43^. Bis-benzimidazole compound (ridinilazole, SMT-19969)^44^ recently entered phase III human clinical trials for the treatment of *Clostridium difficile.*

**Figure 2.**
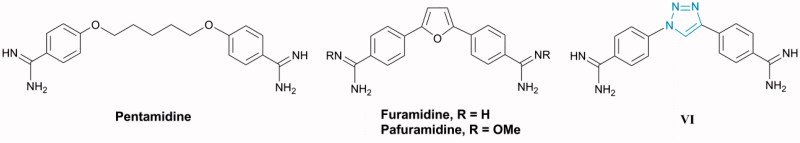
Aromatic amidines and 1,4-diphenyl-1,2,3-triazole amidine VI as anti-HAT agents.

Besides anti-bacterial activity, benzimidazole containing compounds have shown good anti-protozoal potency^45–49^. Human African trypanosomiasis (HAT), also known as sleeping sickness, is a fatal parasitic disease caused by two subspecies of *Trypanosoma brucei*. It has been estimated that over 50 million people are at risk of infection with HAT in more than 30 African countries, and there remains a clear need to develop new, safer, and more affordable agents to combat this fatal infection^50^. The efficacy of diarylamidines, such as pentamidine^51^, berenyl^52^ and its orally active prodrug pafuramidine^53^ (Figure 2), in the treatment of protozoal diseases, especially trypanosomiasis, has been known for many years.

However, current drugs have problems, such as toxicity, poor efficacy, and increasing resistance by the parasites. Although the precise anti-protozoan mechanisms of action of aromatic diamidines have not been fully elucidated, there is considerable evidence that direct interaction with the pathogen genome is important for activity. Recently, a diamidine containing a 1,2,3-triazole ring as central core was synthesised, which displayed better anti-trypanosomal efficacy than melarsoprol, curing all infected mice^54^. It was found that incorporation of different hydrophobic aromatic head groups linked to the rest of the molecule by an amidine moiety improved both anti-bacterial activity and affinity to DNA^27^.

**Figure 3.**
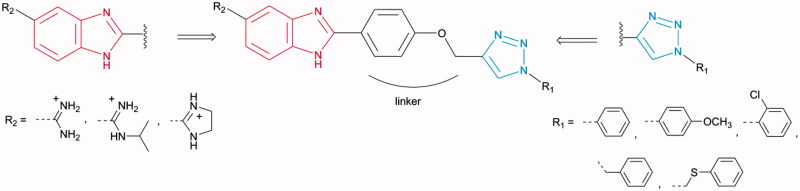
Design and synthesis of amidinobenzimidazoles connected to 1-aryl-substituted 1,2,3-triazole *via* phenoxymethylene unit.

In view of the wide applications of the benzimidazole and 1,2,3-triazole moieties in drug development, and encouraged by the activity profile of both scaffolds^35^ ^,^ ^55^, we synthesised molecules that contained both units attached through a phenoxymethylene linker as the central core, thereby expanding the electronic environment of chemical space (Figure 3).

Targeted compounds were designed to contain a non-substituted amidine, *N*-isopropylamidine, and imidazoline moiety at the C-5 position of the benzimidazole core, as the hydrophilic end, and an aromatic unit at the N-1 position of the 1,2,3-triazole ring, as the hydrophobic end. It was anticipated that selected 5-amidinobenzimidazoles connected to 1-aryl-substituted 1,2,3-triazole would exhibit enhanced affinity for DNA/RNA compared to other aromatic amidines that we have recently studied^56^ ^,^ ^57^. Therefore, interactions between 5-amidinobenzimidazoles **7a**–**7e**, **8a**–**8e**, and **9a**–**9e** and DNA/RNA were assessed and their activities against Gram-positive, Gram-negative, and antibiotic-resistant, as well as their trypanocidal properties, were evaluated.

## Materials and methods

### General

All chemicals and solvents were purchased from Aldrich and Acros. Pre-coated Merck silica gel 60F-254 plates and Fluka (0.063–0.2 mm) silica gel using an appropriate solvent system were employed for thin layer chromatography (TLC) and column chromatography, respectively. Melting points were determined using Kofler micro hot-stage (Reichert, Vienna, Austria). ^1^H and ^13 ^C NMR spectra were recorded on a Varian Gemini 300 and 600 spectrometers. All NMR spectra were recorded in DMSO-d_6_ at 298 K. Mass spectra were recorded on an Agilent 6410 instrument with electrospray interface and triple quadrupole analyser. Microwave-assisted syntheses were carried out in a microwave oven (Milestone start S) at 80 °C and pressure of 1 bar. The ultrasound-assisted reactions were performed in a bath cleaner (Bandelin, Sonorex digital 10 P, Berlin, Germany) with frequency of 35 kHz and power of 1000 W.

### Experimental procedures for the synthesis of compounds

Amidino-substituted *o*-phenylenediamines (**4–6**)^58^, 4-(prop-2-ynyloxy)benzaldehyde (**2**)^59^, 4–(1,2,3-triazol-4-yl)methoxy)benzaldehyde (**3b**)^60^, 4–(1,2,3-triazol-4-yl)methoxy)benzaldehydes (**3a**, **3d**), and 5-amidinobenzimidazoles (**7a**, **7d**, **8a**, **8d**, **9a**, and **9d**)^55^ were prepared according to described procedures.

#### General procedure for the synthesis of compounds 3a–e

The reaction mixture of compound **2**, Cu(0) (0.8 eq), 1 M CuSO_4_ (0.3 eq) and the corresponding azide (1.2 eq) was dissolved in 1 ml DMF and a mixture of *t*-BuOH: H_2_O = 1: 1 (3 ml). Method A: The reaction mixture was stirred under microwave irradiation (300 W) at 80 °C during 1.5 h. Method B: The reaction mixture was placed in an ultrasonic bath cleaner (1000 W, 35 kHz) at 80 °C for 1.5 h. The solvent was removed under reduced pressure and purified by column chromatography with CH_2_Cl_2_.

#### 4-((1-(4-Methoxyphenyl)-1H-1,2,3-triazol-4-yl)methyleneoxy)benzaldehyde (3b)

Compound **3b** was prepared using the above mentioned procedure from **2** (200 mg, 1.15 mmol) and 1-azido-4-methoxybenzene (2.76 ml, 1.38 mmol) to obtain **3b** as white crystals (Method A: 149.3 mg, 42%; Method B: 271.2 mg, 76%; m.p. 127–130 °C) (m.p. lit.^60^ = 126–127 °C). ^1^H NMR (300 MHz, DMSO) δ 9.89 (1H, s, CHO), 8.87 (1H, s, H5′), 7.90 (2H, d, *J* = 8.8 Hz, Ph), 7.81 (2H, d, *J* = 9.1 Hz, Ph), 7.28 (2H, d, *J* = 8.7 Hz, Ph), 7.14 (2H, d, *J* = 9.1 Hz, Ph), 5.36 (2H, s, OCH_2_), 3.83 (3H, s, OCH_3_). ^13 ^C NMR (75 MHz, DMSO) δ 191.55, 162.99, 159.49, 143.08, 131.96, 130.03, 130.00, 123.25, 122.03, 115.35, 115.03, 61.47, 55.68.

#### 4-((1–(2-Chlorophenyl)-1H-1,2,3-triazol-4-yl)methoxy)benzaldehyde (3c)

Compound **3c** was prepared using the above mentioned procedure from **2** (200 mg, 1.15 mmol) and 1-azido-2-chlorobenzene (2.76 ml, 1.38 mmol) to obtain **3c** as white solid (Method A: 194.2 mg, 53%; Method B: 231.67 mg, 64%; m.p. = 127–129 °C). ^1^H NMR (600 MHz, DMSO) δ 9.90 (1H, s, CHO), 8.76 (1H, s, H5′), 7.91 (2H, d, *J* = 8.7 Hz, Ph), 7.79 (1H, dd, *J* = 8.0, 1.2 Hz, Ph), 7.73 (1H, dd, *J* = 7.8, 1.6 Hz, Ph), 7.65 (1H, td, *J* = 7.8, 1.6 Hz, Ph), 7.60 (1H, td, *J* = 7.6, 1.3 Hz, ph), 7.30 (2H, d, *J* = 8.7 Hz, Ph), 5.40 (s, 2H, OCH_2_).^13^C NMR (151 MHz, DMSO) δ 191.84, 163.15, 142.48, 134.54, 132.14, 132.10, 130.84, 130.19, 128.81, 128.74, 128.63, 127.35, 115.54, 61.40.

#### 4-((1-((Phenylthio)methyl)-1H-1,2,3-triazol-4-yl)methoxy)benzaldehyde (3e)

Compound **3e** was prepared using the above mentioned procedure from **2** (200 mg, 1.15 mmol), azidomethyl phenyl sulfide (0.19 ml, 1.38 mmol), Cu (0) (59.8 mg, 0.94 mmol), 1 M CuSO_4_ (0.24 ml, 0.05 mmol) in DMF (1 ml), *t*-BuOH: H_2_O = 1: 1 (4 ml) to obtain **3e** as yellow oil (Method A: 214.3 mg, 57%; Method B: 273.6 mg, 73%). ^1^H NMR (300 MHz, DMSO) δ 9.88 (1H, s, CHO), 8.21 (1H, s, 1H, H5′), 7.87 (2H, d, *J* = 8.8 Hz, Ph), 7.43–7.26 (5H, m, Ph), 7.21 (2H, d, *J* = 8.7 Hz, Ph), 5.96 (2H, s, 2H, CH_2_), 5.26 (2H, s, CH_2_). ^13 ^C NMR (75 MHz, DMSO) δ 191.58, 162.94, 142.57, 132.36, 131.92, 130.68, 129.98, 129.43, 127.90, 124.72, 115.36, 61.33, 51.87.

#### General procedure for the synthesis of compounds 7a–7e, 8a–8e, and 9a–9e

The reaction mixture of 4-triazolylbenzaldehyde derivatives (**3a**–**3e**), *o*-phenylenediamine (**4**, **5,** or **6**) and 40% NaHSO_3_ was dissolved in 15 ml EtOH and stirred under reflux for 6–8 h. After completion of the reaction NaHSO_3_ (aq) was filtered and the reaction mixture was evaporated to dryness. Water was added (5 ml) and the mixture was stirred overnight and filtered. The crude residue was dissolved in HCl saturated MeOH (8–10 ml) and stirred overnight. Addition of ether resulted in precipitation of products **7a**–**7e**, **8a**–**8e**, and **9a**–**9e**. Solid was collected by filtration, washed with anhydrous ether, and dried under vacuum.

#### 2–(4-((1–(4-Methoxyphenyl)-1H-1,2,3-triazol-4-yl)methoxy)phenyl)-1H-benz[d]imidazole-5-carboximidamide dihydrochloride (7b)

Compound **7b** was prepared using the above described method from **3b** (200 mg, 0.65 mmol) and **4** (87.39 mg, 0.58 mmol) to obtain **7b** as white powder (122.7 mg, 53%, m.p. = 195–197 °C). ^1^H NMR (600 MHz, DMSO) δ 9.35 (2H, s, NH), 8.94 (2H, s, NH), 8.90 (1H, s, H5′), 8.26 (2H d, *J* = 8.2 Hz, Ph), 8.15 (1H, s, H4), 7.84–7.80 (3H, m, Ph; H7), 7.71 (1H, d, *J* = 8.0 Hz, H6), 7.34 (2H, d, *J* = 8.7 Hz, Ph), 7.15 (2H, d, J = 9.0 Hz, Ph), 5.37 (2H, s, OCH_2_), 3.84 (3H, s, OCH_3_). ^13 ^C NMR (75 MHz, DMSO) δ 165.85, 160.58, 159.42, 153.78, 143.21, 140.84, 134.73, 129.97, 129.13, 123.14, 122.69, 121.93, 120.61, 115.55, 114.97, 61.34, 55.63. MS (ESI, *m/z*) 440.1 [M + H]^+^. Anal. calcd. for C_24_H_21_N_7_O_2_ × 2 HCl ×2.5 H_2_O (Mr = 557.44): C 51.71, H 5.06, N 17.59; found: C 51.60, H 4.72, N 17.34%.

#### 2–(4-((1–(2-Chlorophenyl)-1H-1,2,3-triazol-4-yl)methoxy)phenyl)-1H-benz[d]imidazole-5-carboximidamide dihydrochloride (7c)

Compound **7c** was prepared using the above described method from **3c** (200 mg, 0.64 mmol) and **4** (96.51 mg, 0.64 mmol) to obtain white powder **7c** (210.3 mg, 58%, m.p. = 176–177 °C). ^1^H NMR (300 MHz, DMSO) δ 9.36 (2H, s, NH), 8.96 (2H, s, NH), 8.77 (1H, s, 1H, H5′), 8.28 (2H, d, *J* = 8.8 Hz, Ph), 8.16 (1H, s, H4), 7.88–7.55 (6H, m, Ph; H5; H6), 7.36 (2H, d, *J* = 8.9 Hz, Ph), 5.40 (2H, s, OCH_2_). ^13 ^C NMR (151 MHz, DMSO) δ 165.62, 161.33, 152.72, 142.25, 134.41, 131.84, 131.26, 130.60, 129.84, 128.51, 127.23, 123.76, 123.22, 122.24, 115.90, 115.70, 114.82, 61.23. MS (ESI, *m/z*) 444.0 [M + H]^+^. Anal. calcd. for C_23_H_18_ClN_7_O × 2 HCl ×3 H_2_O (Mr = 570.86): C 48.39, H 4.59, N 17.17; found: C 48.11, H 4.47, N 17.38%.

#### 2–(4-((1-((Phenylthio)methyl)-1H-1,2,3-triazol-4-yl)methoxy)phenyl)-1H-benz[d]imidazole-5-carboximidamide dihydrochloride (7e)

Compound **7e** was prepared using the above described method from **3e** (200 mg, 0.64 mmol) and **4** (96.51 mg, 0.64 mmol) to obtain **7e** as white powder (324.8 mg, 90%, m.p. = 173–176 °C). ^1^H NMR (300 MHz, DMSO) δ 9.46 (2H, s, NH), 9.08 (2H, s, NH), 8.34 (2H, d, *J* = 8.9 Hz, Ph), 8.25 (1H, s, H5′), 8.20 (1H, d, *J* = 1.1 Hz, H4), 7.89 (2H, t, *J* = 7.8 Hz, Ph), 7.80 (1H, dd, *J* = 8.6 Hz, H6), 7.45–7.29 (6H, m, H7; Ph), 5.99 (2H, s, CH_2_), 5.30 (2H, s, CH_2_).^13^C NMR (75 MHz, DMSO) δ 165.70, 161.12, 153.11, 142.62, 139.17, 132.38, 131.84, 130.59, 129.59, 129.38, 127.82, 124.66, 123.55, 122.98, 115.72, 115.30, 61.27, 51.80. MS (ESI, *m/z*) 456.1 [M + H]^+^. Anal. calcd. for C_24_H_21_N_7_OS ×2 HCl ×1.7 H_2_O (Mr = 559.09): C 51.56, H 4.76, N 17.54; found: C 51.80, H 4.68, N 17.22%.

#### N-isopropyl-2–(4-((1–(4-methoxyphenyl)-1H-1,2,3-triazol-4-yl)methoxy)phenyl)-1H-benz[d]imidazole-5-carboximidamide dihydrochloride (8b)

Compound **8b** was prepared using the above described method from **3b** (200 mg, 0.65 mmol) and **5** (115.2 mg, 0.65 mmol) to obtain **8b** as brown powder (225.8 mg, 58%, m.p. = 188–191 °C). ^1^H NMR (300 MHz, DMSO) δ 9.66 (1H, d, *J* = 7.7 Hz, NH), 9.51 (1H, s, NH), 9.08 (1H, s, NH), 8.92 (1H, s, H5′), 8.39 (2H, d, *J* = 8.7 Hz, Ph), 8.08 (1H, s, H4), 7.88 (1H, d, *J* = 8.5 Hz, H7), 7.83 (2H, d, *J* = 9.0 Hz, Ph), 7.69 (1H, d, *J* = 7.6 Hz, H6), 7.39 (2H, d, *J* = 8.8 Hz, Ph), 7.15 (2H, d, *J* = 9.0 Hz, Ph), 5.39 (2H, s, CH_2_), 4.15–4.02 (1H, m, CH), 3.84 (3H, s, OCH_3_), 1.32 (6H, d, *J* = 6.3 Hz, CH_3_CHCH_3_).^13^C NMR (151 MHz, DMSO) δ 162.18, 161.13, 159.50, 152.96, 143.20, 130.02, 129.64, 124.28, 124.21, 123.74, 123.30, 122.03, 115.77, 115.36, 115.05, 61.43, 55.71, 45.25, 21.38. MS (ESI, *m/z*) 482.1 [M + H]^+^. Anal. calcd. for C_27_H_27_N_7_O_2_ × 2 HCl ×2.6 H_2_O (Mr = 601.32): C 53.93, H 5.73, N 16.30; found: C 53.62, H 5.71, N 16.39%.

#### N-isopropyl-2–(4-((1–(2-chlorophenyl)-1H-1,2,3-triazol-4-yl)methoxy)phenyl)-1H-benz[d]imidazole-5-carboximidamide dihydrochloride (8c)

Compound **8c** was prepared using the above described method from **3c** (200 mg, 0.64 mmol) and **5** (101.0 mg, 0.57 mmol) to obtain **8c** as white powder (105.5 mg, 28%, m.p. = 210–213 °C). ^1^H NMR (300 MHz, DMSO) δ 9.58 (1H, d, *J* = 8.2 Hz, NH), 9.43 (1H, s, NH), 8.99 (1H, s, NH), 8.78 (1H, s, H5′), 8.30 (2H, d, *J* = 8.6 Hz, Ph), 8.04 (1H, s, H4), 7.87–7.57 (6H, m, H7; H6; Ph), 7.37 (2H, d, *J* = 8.4 Hz, Ph), 5.40 (2H, s, OCH_2_), 4.13–4.00 (1H, m, CH), 1.31 (6H, d, *J* = 6.1 Hz, CH_3_CHCH_3_). ^13 ^C NMR (151 MHz, DMSO) δ 162.41, 160.38, 153.72, 142.46, 134.42, 131.84, 130.61, 128.93, 128.57, 128.55, 128.47, 127.09, 123.10, 127.09, 122.58, 120.24, 116.13, 115.53, 61.14, 45.06, 21.31. MS (ESI, *m/z*) 486.1 [M + H]^+^. Anal. calcd. for C_26_H_24_ClN_7_O × 2 HCl ×2.3 H_2_O (Mr = 600.33): C 52.02, H 5.14, N 16.33; found: C 52.22, H 5.03, N 16.59%.

#### N-isopropyl-2–(4-((1-(phenylthiomethyl)-1H-1,2,3-triazol-4-yl)methoxy)phenyl)-1H-benz[d]imidazole-5-carboximidamide dihydrochloride (8e)

Compound **8e** was prepared using the above described method from **3e** (200 mg, 0.61 mmol) and **5** (108.9 mg, 0.61 mmol) to obtain **8e** as yellow powder (73.1 mg, 21%, m.p. = 152–154 °C). ^1^H NMR (600 MHz, DMSO) δ 9.66 (1H, d, *J* = 7.5 Hz, NH), 9.51 (1H, s, NH), 9.09 (1H, s, NH), 8.39 (2H, d, *J* = 7.9 Hz, Ph), 8.25 (1H, s, H5′), 8.08 (1H, s, H4), 7.87 (1H, d, *J* = 8.3 Hz, H7), 7.68 (1H, d, *J* = 8.5 Hz, H6), 7.40 (2H, d, *J* = 7.5 Hz, Ph), 7.36–7.15 (5H, m, Ph), 5.98 (2H, s, CH_2_), 5.29 (2H, s, CH_2_), 4.24–3.99 (1H, m, CH), 1.31 (6H, d, *J* = 6.4 Hz, CH_3_CHCH_3_).^13^C NMR (75 MHz, DMSO) δ 162.93, 160.52, 154.42, 145.37, 143.19, 132.82, 130.98, 129.76, 129.09, 128.18, 124.99, 123.19, 122.71, 122.05, 115.86, 61.55, 52.18, 45.44, 21.75. MS (ESI, *m/z*) 498.1 [M + H]^+^. Anal. calcd. for C_27_H_27_N_7_OS ×2 HCl ×0.3 H_2_O (Mr = 575.95): C 56.31, H 5.18, N 17.02; found: C 56.33, H 5.37, N 17.28%.

#### 5–(4,5-Dihydro-1H-imidazol-2-yl)-2–(4-((1–(4-methoxyphenyl)-1H-1,2,3-triazol-4-yl)methoxy)phenyl)-1H-benz[d]imidazole dihydrochloride (9b)

Compound **9b** was prepared using the above described method from **3b** (200 mg, 0.65 mmol) and **6** (114.9 mg, 0.65 mmol) to obtain **9b** as yellow powder (279.1 mg, 70%, m.p. = 197–199 °C). ^1^H NMR (300 MHz, DMSO) δ 10.68 (2H, s, NH), 8.91 (1H, s, H5′), 8.37 (1H, s, H4), 8.32 (2H, d, *J* = 8.3 Hz, Ph), 7.88 (1H, d, *J* = 4.3 Hz, H7), 7.82 (2H, d, *J* = 8.5 Hz, Ph), 7.35 (2H, d, *J* = 8.1 Hz, Ph), 7.28 (1H, d, *J* = 8.3 Hz, H6), 7.15 (2H, d, *J* = 8.1 Hz, Ph), 5.37 (2H, s, OCH_2_), 4.03 (4H, s, CH_2_ CH_2_), 3.83 (3H, s, OCH_3_).^13^C NMR (151 MHz, DMSO) δ 165.49, 160.68, 159.51, 154.27, 143.30, 143.09, 136.43, 131.97 (C4), 130.01 (Ph-q), 129.27 (Ph), 123.21 (C6), 123.00 (C5′), 122.04 (Ph), 120.62 (C5), 116.05, 115.63, 115.37, 115.06, 61.38, 55.72, 44.44. MS (ESI, *m/z*) 466.1 [M + H]^+^. Anal. calcd. for C_26_H_23_N_7_O_2_ × 2 HCl ×0.9 H_2_O (Mr = 554.65): C 56.30, H 4.87, N 17.68; found: C 56.04, H 4.72, N 17.97%.

#### 5–(4,5-Dihydro-1H-imidazol-2-yl)-2–(4-((1–(2-chlorophenyl)-1H-1,2,3-triazol-4-yl)methoxy)phenyl)-1H-benz[d]imidazole dihydrochloride (9c)

Compound **9c** was prepared using the above described method from **3c** (200 mg, 0.64 mmol) and **6** (112.9 mg, 0.64 mmol) to obtain **9c** as red powder (115.3 mg, 30%, m.p. = 194–196 °C). ^1^H NMR (600 MHz, DMSO) δ 10.82 (2H, s, NH), 8.81 (1H, s, H5′), 8.45 (1H, s, H4), 8.40 (2H, d, *J* = 8.6 Hz, Ph), 7.98 (1H, d, *J* = 8.5 Hz, H7), 7.93 (1H, d, *J* = 8.3 Hz, H6), 7.81 (1H, dd, *J* = 8.0, 1.2 Hz, Ph), 7.75 (1H, dd, *J* = 7.8, 1.6 Hz, Ph), 7.67 (1H, td, *J* = 7.8, 1.6 Hz, Ph), 7.62 (1H, td, *J* = 7.7, 1.3 Hz, Ph), 7.40 (2H, d, *J* = 8.9 Hz, Ph), 5.43 (2H, s, OCH_2_), 4.04 (4H, s, CH_2_ CH_2_).^13^C NMR (75 MHz, DMSO) 165.20, 161.07, 153.69, 142.40, 138.84, 136.92, 134.44, 131.90, 130.66, 130.15, 129.62, 128.63, 128.52, 127.21, 123.57, 119.46, 116.66, 116.38, 115.69, 61.24, 44.42. MS (ESI, *m/z*) 470.1 [M + H]^+^. Anal. calcd. for C_25_H_20_ClN_7_O × 2 HCl ×1.9 H_2_O (Mr = 577.08): C 52.03, H 4.51, N 16.99; found: C 52.31, H 4.66, N 16.63%.

#### 5–(4,5-Dihydro-1H-imidazol-2-yl)-2–(4-((1-(phenylthiomethyl)-1H-1,2,3-triazol-4-yl)methoxy)phenyl)-1H-benz[d]imidazole dihydrochloride (9e)

Compound **9e** was prepared using the above described method from **3e** (200 mg, 0.61 mmol) and 6 (108.9 mg, 0.61 mmol) to obtain **9e** as brown powder (149.4 mg, 41%, m.p. = 162–164 °C). 1H NMR (300 MHz, DMSO) δ 10.57 (2H, s, NH), 8.34–8.18 (4H, m, H5′; H4; Ph), 7.85 (2H, s, H7; H6), 7.44–7.21 (7H, m, Ph), 5.97 (2H, s, CH2), 5.26 (2H, s, CH2), 4.03 (4H, s, CH2CH2).13C NMR (151 MHz, DMSO) δ 165.13, 162.84, 160.80, 153.75, 142.60, 132.38, 131.79, 130.48, 129.86, 129.40, 129.32, 127.72, 124.63, 116.37, 115.52, 115.23, 61.17, 51.69, 44.33. MS (ESI, *m/z*) 482.0 [M + H]+. Anal. calcd. for C26H23N7OS ×2 HCl ×2.2 H_2_O (Mr = 594.14): C 52.56, H 4.99, N 16.50; found: C 52.64, H 5.12, N 16.29%.

### Spectroscopic experiments

#### Polynucleotides

Polynucleotides were purchased as follows: polyG–polyC, polyA–polyU (Sigma-Aldrich, St. Louis, MO), calf thymus *ct*DNA (Aldrich). Polynucleotides were dissolved in PBS buffer, *I* =  0.05 mol dm^−3^, pH 7.0. The calf thymus *ct*DNA was additionally sonicated and filtered through a 0.45 mm filter. The polynucleotide concentration was spectroscopically determined as the concentration of nucleobases^62^.

#### UV–Vis spectroscopy

All UV–Vis absorbance measurements were conducted on a Perkin Elmer Lambda 25 spectrophotometer (Perkin Elmer, Waltham, MA). A quartz cell with a 1 cm path length was used for all absorbance studies. Compound stock solutions were 1 mM. The DNA/RNA at increasing ratios was then titrated into the compound buffer solution (1.48 × 10^−5 ^mol dm^−3^) and the corresponding absorption spectra were recorded under the same conditions. All data were graphed and analysed using Origin software version 9.0 (OriginLab Corporation, Northampton, MA). The stability constants (Ks) and [bound compound]/[polynucleotide phosphate] ratios (*n*) were calculated according to the Scatchard Equation^63^ ^,^ ^64^. Values for Ks and *n* given in Table 1 all have satisfying correlation coefficients (0.99).

**Table 1.** Hypochromic effects (H/%)^a^, binding constants (log *K_s_*)^b^ and ratios *n* ^c^ ([compound]/[polynucleotide phosphate]) calculated from the UV–Vis titrations of compounds with ds-DNA/RNA (PBS, *I* = 0.015 M, and pH = 7).

Compound	*ct*DNA			polyA-polyU			polyC-polyG		
	H/%^c^	log *Ks*	*n*	H/%^c^	log *Ks*		H/%^c^	log *Ks*	*n*
**7a**	7.8	6.76	0.27	19.3	5.24	0.31	5.26	6.44	0.40^d^
**7b**	22.1^e^	–	–	27.9^e^	–	–	13.7	6.27	0.73
**7c**	36.3	6.24	0.58	38.9	5.21	0.18	4.82^e^	–	–
**7d**	40.4	5.78	0.59	32.5	5.94	0.11	4.96	6.56	0.36
**7e**	38.2	5.33	0.61	31.7	6.20	0.29	5.79	5.89	0.69
**8a**	41.0	6.17	0.64	46.5	5.87	0.24	5.86	6.17	0.4^d^
**8b**	29.7	6.06	0.62^f^	57.8	5.27	0.23	5.05	6.39	0.39
**8c**	33.8	5.64	0.69	50.4	6.00	0.19	5.31	6.43	0.36
**8d**	41.2	5.93	0.47	53.8	6.13	0.30	6.13	5.54	0.40^d^
**8e**	44.2	6.39	0.36^f^	47.2	5.96	0.30	5.96	7.20	0.28
**9a**	25.7^e^	–	–	32.7^e^	–	–	–	6.21	0.40^d^
**9b**	18.9^e^	–	–	31.6^e^	5.46	0.30^d^	5.46^e^	–	–
**9c**	11.7	6.43	0.35	29.3	5.38	0.29	5.34	6.43	0.36
**9d**	24.6	5.84	0.75	55.5^e^	5.89	0.30^d^	5.89	6.59	0.41
**9e**	41.1	6.21	0.12	31.6	5.36	0.26	5.21	4.29	0.4^d^

a Hypochromic effect calculated by Scatchard equation for compounds; *H* = (Abs(compound) –Abs(complex))/Abs(compound) × 100.

b Titration data were processed according to the Scatchard Equation^63^ ^,^ ^64^.

c Accuracy of *n* ± 10–30%, consequently log*K_s_* values vary in the same order of magnitude.

d *n* = fix.

e Hypochromic effect calculated from experimental data.

f Mixed binding mode and binding constants were calculated in range *r* ≥ 0.1.

−: changes were too small for accurate calculation of binding constants.

#### Thermal melting (T_m_)

*T*_m_ experiments were conducted with a Perkin Elmer Lambda 25 spectrophotometer in 1 cm quartz cuvettes. The absorbance of the DNA/RNA-compound complex was monitored at 260 nm as a function of temperature. The absorbance of the ligands was subtracted from every curve, and the absorbance scale was normalised. The Δ*T*_m_ values were calculated by subtracting *T*_m_ of the free nucleic acid from *T*_m_ of the complex. Every reported Δ*T*_m_ value was the average of at least two measurements. The error of Δ*T*_m_ is ±0.5 °C. All data were graphed and analysed using Origin software version 9.0.

#### Circular dichroism (CD)

The CD spectra of DNA/RNA (concentration in cuvette 2 × 10^−5 ^M) were recorded with a JASCO J-800 spectrometer (JASCO UK Ltd., Dunmow, United Kingdom) at different ratios *r* = 0.1, 0.3, 0.5, and 0.7 (*r* = [compound]/[polynucleotide]) at 25 °C in aqueous buffer solution (pH = 7, PBS, and *I* = 0.05 mol dm^−3^). Titrations were carried out by addition of aliquots of 1 mM stock solutions of the relevant compound (at increasing ratios) to the buffered polynucleotide (DNA/RNA) solution in a 1 cm quartz cuvette and scanned over a wavelength range 220–450 nm. All data were graphed and analysed using Origin software version 9.0.

### Biological evaluations

#### Anti-bacterial screening

The compounds were evaluated for their *in vitro* anti-bacterial activity against Gram-positive bacteria: *S. aureus* (ATCC 25923), MRSA, methicillin-sensitive *S. aureus* (MSSA), *E. faecalis,* vancomycin-resistant *E. faecium* (VREF), and Gram-negative bacteria: *E. coli* (ATCC 25925), *P. aeruginosa* (ATCC 27853), *A. baumannii* (ATCC 19606) and ESBL-producing *K. pneumoniae* (ATCC 27736). Standard broth microdilution method as recommended in guidelines of Clinical and Laboratory Standards Institute^61^ ^,^ ^65^ ^,^ ^66^ was applied and the minimum inhibitory concentration (MIC) of compounds was tested. In short, testing was performed in U-bottomed 96-well sterile plastic microdilution trays (Falcon 3077, Becton Dickinson Labware, Franklin Lakes, NJ) in cation (Ca^2+^ and Mg^2+^) adjusted Mueller–Hinton broth medium (Becton Dickinson and Co., Cockeysville, MD). All testings were performed in triplicate.

#### Anti-trypanosomal screening and cytotoxicity assays

Bloodstream form *T. brucei* (strain 221) were cultured in modified Iscove’s medium, as outlined^67^ and trypanocidal assays were performed using 96-well microtitre plates. The compound concentrations that inhibited growth by 50% (IC_50_) and 90% (IC_90_) were determined. Parasites were sub-cultured at 2.5 × 10^4^ ml^−1^, compounds were added at range of concentrations, and the plates incubated at 37 °C. Resazurin was added after 48 h, the plates incubated for a further 16 h, and then read in a Spectramax plate reader (Molecular Devices Corporation, San Jose, CA). The data were analysed using GraphPad Prism (GraphPad, La Jolla, CA). Each drug concentration was tested in triplicate.

Cytotoxicity against mammalian cells was also assessed using microtitre plates. Briefly, L6 cells (a rat myoblast line) were seeded at 1 × 10^4^ ml^−1^ in 200 µl of growth medium containing different compound concentrations. The plates were then incubated for 6 d at 37 °C and 20 µl resazurin added to each well. After a further 8 h incubation, the fluorescence was determined using a Spectramax plate reader, as outlined above.

## Results and discussion

### Chemistry

**Scheme 1.**
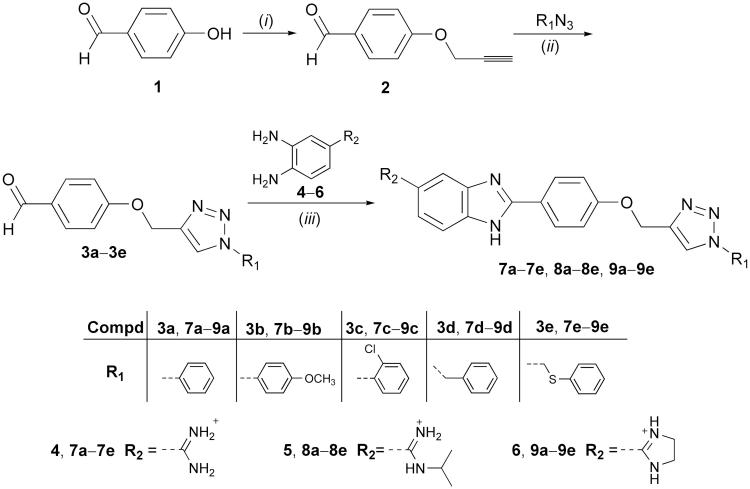
Synthesis of 2,5-disubstituted benzimidazoles. (i): propargyl bromide, K_2_CO_3_, EtOH, reflux; (ii): corresponding azides, CuSO_4_, Cu(0), DMF, t-BuOH: H_2_O = 1: 1, 80 °C; (iii): *o*-phenylenediamine **(4–6)**, NaHSO_3_, EtOH, reflux; HCl/MeOH, room temperature.

1,2,3-Triazole-linked 5-amidinobenzimidazoles **7a**–**7e**, **8a**–**8e**, and **9a**–**9e** are synthesised as outlined in Scheme 1. 4-Hydroxybenzaldehyde was propargylated to give 4-(prop-2-ynyloxy)benzaldehyde (**2**), which subsequently *via* the regioselective Cu(I) catalysed cycloaddition with aromatic azides resulted in 4–(1,2,3-triazol-1-yl)benzaldehyde derivatives (**3a**–**3e**) comprising an *N*-1-aryl-substituted 1,2,3-triazole subunit. An efficient and environmentally benign synthetic protocol^68^, applying microwave and ultrasound irradiation, was used in the synthesis of **3a**–**3e**. The efficiency of both ultrasound and microwave conditions were compared and indicated that ultrasound-assisted syntheses of **3a**–**3e** resulted in higher yields than those of microwave-assisted reactions. Amidino-substituted 1,2-phenylenediamines (**4–6**) that were used for the synthesis of the target 5-amidinobenzimidazoles **7a**–**7e**, **8a**–**8e**, and **9a**–**9e** were synthesised from the corresponding nitrile by the Pinner method^58^. 4-Amidino 1,2-phenylenediamines (**4–6**) reacted with the bisulfite adduct of the 4–(1,2,3-triazol-1-yl)benzaldehyde derivatives (**3a**−**e**) to produce amidine (**7a**–**7e**), *N*-isopropylamidine (**8a**–**8e**), and imidazoline-substituted (**9a**–**9e**) benzimidazole derivatives^69^.

### Spectroscopic characterisation of compounds

5-Amidinobenzimidazole derivatives **7a**–**7e**, **8a**–**8e**, and **9a**–**9e** were synthesised and characterised by UV–Vis spectroscopy. UV–Vis spectra displayed two absorption maxima at around 260 and 315 nm (Table S1, Supporting Information). Absorbancies of solutions were proportional to their concentrations up to 1 × 10^−4^ moldm^−3^, indicating that there is no significant intermolecular stacking that could give rise to hypochromic effects. Furthermore, the UV–Vis spectra of **7a**–**7e**, **8a**–**8e**, and **9a**–**9e** revealed negligible temperature dependent changes (25–90 °C) and excellent reproducibility upon cooling to 25 °C. The results showed that all evaluated compounds were stable and suitable for further spectroscopic and biological investigations.

### Interactions with double-stranded polynucleotides

#### Spectrophotometric titrations of compounds with ds-DNA/RNA

UV–Vis absorption spectroscopy is simple, widely used and one of the most effective methods for detecting the interaction of small molecules with DNA. In general, these interactions and the subsequent formation of a new complex leads to changes in UV–Vis spectra^70^. Therefore, UV–Vis spectroscopy was applied to investigate the interaction of compounds **7a**–**7e**, **8a**–**8e**, and **9a**–**9e** with ds-DNA/RNA. UV–Vis titration with *ct*DNA showed a hypochromic effect indicating the disappearance of free molecule and the formation of a new compound-DNA species (Figure S1, Supporting Information). The hypochromic effect (12–44%) was accompanied by a small bathochromic shift (Δ*λ* = 3–9 nm) that was found to originate from the stabilisation of DNA secondary structure due to the interaction with small molecules^71^.

To assess the sequence selectivity of the compounds, the experiment was repeated with ds-RNA polynucleotides (polyA-polyU and polyC-polyG). The addition of polyA-polyU in most cases led to hypochromic (19–58%) and small bathochromic (2–11 nm) changes in the visible absorption spectra as a result of complex formation. Absorption spectra obtained by adding aliquots of polyC-polyG to the compound solutions were recorded until saturation was achieved. In general, it was observed that addition of polyC-polyG resulted in a pronounced decrease of UV–Vis absorption maxima at 300–320 nm (27–50%), followed by small bathochromic shifts (Δλ = 2–7 nm). No further studies were conducted with compounds whose UV–Vis spectra showed minimal changes (Δ*A* < 0.08 at *r* = 1–0.1) during titration with DNA/RNA polynucleotides. It can be inferred that these compounds interact with polynucleotides only through a very weak electrostatic and external mode (Table 1). During titration with polyA-polyU, a clear isosbestic point was observed in UV–Vis spectra of **7a** and **9c**, pointing to the formation of one dominant type of complex.

The binding constants log*K*s and the density of the binding sites *n* were calculated using Scatchard plot analysis. In addition, the binding constants *K*s for compounds **8b** and **8e** were calculated only for titration data taken at the *r* ≥ 0.1, because below that ratio changes in absorption maxima were too small for accurate calculation (ΔA ≤ 0.04) (Table 1). The binding constants *K*s and ratios *n* obtained by processing UV–Vis titration data using the Scatchard equation are summarised in Table 1.

#### Thermal denaturation experiments

Thermal melting enables the rapid qualitative evaluation of the relative binding affinities of the compounds towards selected polynucleotides (Table 2)^72^ ^,^ ^73^. The melting temperature (*T*_m_) is defined as the differences between the melting temperatures of free polynucleotides and their complexes with small non-covalently bound molecules. The correlation between binding constant and the increase of *T*_m_ was found to be quite complex, because the number of binding sites, positive charge of compounds, potential cooperativity, and the affinity for the unfolded polynucleotide have also to be taken into account^74^.

**Table 2.** Δ*T*_m_ values (°C) of studied ds-polynucleotides upon addition of compounds **7a**, **7c**–**7e**, **8a**–**8e**, and **9b**–**9e** at different ratio *r* ^b^ (PBS and pH = 7)^a^.

Compound	*ct*DNA			polyA-polyU		
	0.3	0.5	0.7	0.1	0.3	0.5
**7a**	2.46	4.84	4.69	1.74	1.74	2.53
**7c**	2.35	3.20	3.77	0.20	2.72	–^d^
**7d**	3.12	3.86	3.62	2.06	1.14	1.709.05^c^
**7e**	2.19	2.62	2.66	0.39	1.11	1.11
					12.58^c^	15.04^c^
**8a**	3.35	3.94	4.25	0.32	0.64	1.11
**8b**	4.03	3.96	4.47	1.17	1.32	1.56
					6.38^c^	8.49^c^
**8c**	3.40	3.89	4.39	0.76	0.58	1.12
**8d**	1.76	3.32	3.63	0.91	0.51	0.72
**8e**	2.22	1.72	1.71	0.52	0.23	0.26
**9b**	–	–	–	2.49	2.67	2.92
					36.08^c^	40.93^c^
**9c**	3.55	3.31	3.80	2.85	4.2713.99^c^	5.5317.73^c^
**9d**	3.31	3.52	2.60	1.26	1.42	2.06
					10.30^c^	13.29^c^
**9e**	2.87	3.47	3.60	1.11	0.47	0.47
					12.46^c^	13.89^c^

a All values are averaged from at least two measurements. Error in Δ*T*_m_: ±0.5 °C.

b *r* = [compound]/[polynucleotide].

c Biphasic melting curve, values for both melting midpoints given when possible.

d Not possible to determine due to the lack of melting midpoint.

Denaturation experiments were carried out at different amounts of the compounds (*r* = 0.1, 0.3, 0.5, and 0.7 eq; *r* = [compound]/[polynucleotide]) with *ct*DNA and polyA-polyU. The results of the denaturation experiments are listed in Table 2.

Generally, results correlated with those of UV–Vis experiments. Strong non-linear dependence of Δ*T*_m_ values on the ratio *r* was revealed, suggesting saturation of binding sites at *r* = 0.5–0.7 (for **7c**–**7d**, **8a**–**8e**, and **9d**), *r* = 0.3–0.5 (for **7a**, **9c**, and **9e**), in good accordance with the calculated values presented in Table 1. Results showed that compounds **8a**–**8e** stabilised *ct*DNA slightly better than compounds **7a**–**7e** and **9a**–**9e**. Biphasic curves for interactions of compounds **7e**, **8b**, and **9b**–**9e** with polyA-polyU at higher ratios *r* indicated additional binding modes. The above-mentioned compounds have monophasic curves at *r* ≤ 0.3, which together with results on UV–Vis titration confirmed intercalation as the dominant binding mode, while above that, ratio biphasic curves indicated agglomeration of compounds along the polynucleotide chains.

#### Circular dichroism (CD) experiments

**Figure 4.**
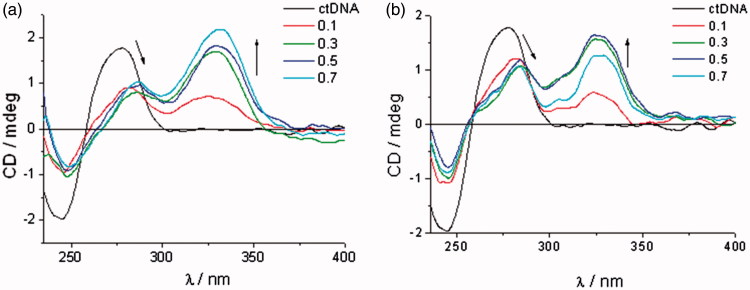
Induced CD spectra of compound **7a** (a) and compound **8c** (b) with *ct*DNA (*r* = 0–0.7).

CD spectroscopy has been extensively employed for the investigation of small molecule–polynucleotide (DNA/RNA) interactions^75^ ^,^ ^76^. Binding of achiral small molecules within the chiral DNA/RNA helix results in an induced CD spectrum (ICD)^77^ ^,^ ^78^. The appearance of ICD bands upon titration (*r* = 0.1–0.7) at *λ* > 300 nm was used to estimate the orientation of the chromophore in the *ct*DNA/RNA binding site and for the determination of binding mode. *ct*DNA features approximately 40% GC and 60% AT base pairs and adopts a B-helix with a narrow, deep, well-accessible minor groove and a rather broad, and shallow major groove. The two RNA polymers, polyA-polyU and polyG-polyC, form a typical A-helix with a broad minor and narrow major groove. The main difference among ds-RNAs is the presence of the amino group at N-2 in guanine which protrudes into the grooves and thereby may influence the affinity and binding mode of compounds being studied. The addition of the compounds being investigated resulted in a decrease of the ds-DNA/RNA CD bands (*λ* = 220–400 nm, S2). Observed changes in the intensity of CD bands for ds-DNA/RNA indicated the partial disruption of the polynucleotide helical chirality upon binding of a small molecule. The addition of the compounds **7a**, **7c–7e**, **8a–8e**, and **9c–9d** in solution with *ct*DNA generated strong, positive ICD signals in the range of 300–350 nm (Figures 4 and S2, Supporting Information). This may arise due to groove binding being the dominant binding mode for this class of compounds^79^ ^,^ ^80^.

**Figure 5.**
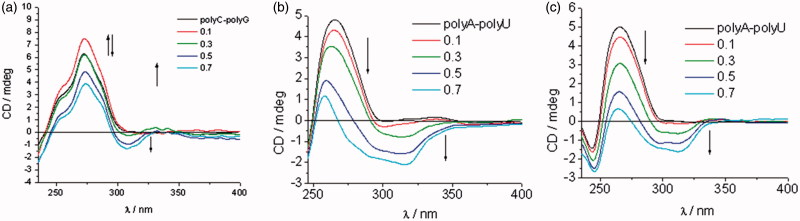
ICD spectra of RNA polynucleotides with 5-amidinobenzimidazoles with *p*-methoxyphenyl-1,2,3-triazole unit: compound **7b** (a), compound **8b** (b), and compound **9b** (c).

ICD spectra of polyA-polyU and polyC-polyG with the addition of evaluated compounds, except for **7a**, **8a**–**8e**, and **9d**, showed a decrease of CD band in the range of 220–300 nm, followed by appearance of new negative signal at >300 nm (Figures 5 and S2, Supporting Information). This indicates that intercalation is the dominant binding mode. Compounds **7b**–**9b** showed higher affinity for dsRNA than *ct*DNA. While **8b** and **9b** bound to polyA-polyU, **7b** showed higher affinity for polyC-polyG (Figures 5 and S2, Supporting Information).

ICD spectra of **8b** and **9b**, in the presence of polyA-polyU, showed an intense increase of signal above *r* = 0.3, while ICD spectra of **7b** with polyC-polyG showed a weaker intercalation signal above *r* = 0.5. This is in agreement with the results obtained by UV–Vis and thermal melting methods. Minimal changes of the intensity of the CD bands of polyC-polyG upon titration with compounds **7a, 8a–8e**, and **9d**, suggest a non-specific binding mode. Most probably compounds bind on the outside of the polyC-polyG polynucleotide. The intensity of negative ICD bands in polyA-polyU ICD spectra was also observed to be more intense than those in polyC-polyG spectra obtained with the same compound.

### Biological evaluations

#### In vitro anti-bacterial activity

The *in vitro* anti-bacterial activity of 5-amidinobenzimidazoles **7a**–**7e**, **8a**–**8e**, and **9a**–**9e** was tested against Gram-positive bacteria including *S. aureus* (ATCC 25923), *Enterococcus faecalis* (ATCC 29212), and Gram-negative bacteria including *E. coli* (ATCC 25925), *K. pneumoniae* (ATCC 700803), *P. aeruginosa* (ATCC 27853), and *Acinetobacter baumannii* (ATCC 19606). The MICs were determined and compared with those of the antibiotics ceftazidime, ciprofloxacin, ampicillin, and gentamicin (Table S2, Supporting Information).

Generally, compounds showed better activities against Gram-positive than Gram-negative bacteria. Only 5-amidinobenzimidazoles **7a**, **7d**, and **7e** proved to be active against three Gram-negative strains, particularly amidinobenzimidazole **7d,** which has an *N*-1-benzyl substituent. The type of amidino moiety in 5-benzimidazole had impact on the anti-bacterial activities, with non-substituted amidinobenzimidazoles **7a**–**7e** having the highest overall activities (Table S2, Supporting Information). Compounds that exhibited anti-bacterial activities with MIC <256 µg/ml were evaluated against antibiotic resistant Gram-positive clinical strains, such as MRSA, MSSA and VREF (Table 3) and Gram-negative clinical strains including extended-spectrum *β*-lactamase (ESBL)-producing *E. coli*, *K. pneumoniae*, and *P. aeruginosa* (Table 4).

**Table 3.** Anti-bacterial activity of selected compounds against antibiotic-resistant Gram-positive clinical strains.

Compound	MIC (µg/ml)		
	*S. aureus*	*S. aureus*	*E. faecium*
	MRSA	MSSA	VRE
**7a**	16	32	32
**7b**	–	–	256
**7c**	16	32	256
**7d**	16	32	32
**7e**	32	128	64
**8a**	8	128	128
**8b**	16	64	128
**8c**	8	16	32
**8d**	32	64	64
**8e**	8	64	64
**9a**	128	128	128
**9b**	256	256	256
**9c**	8	128	64
**9d**	64	64	64
**9e**	64	64	32
Ampicillin	4	1	1
Gentamicin	0.25	0.125	0.25

**Table 4.** Anti-bacterial activity of selected compounds against antibiotic-resistant Gram-negative clinical strains.

Compound	MIC (µg/ml)		
	*E. coli*	*K. pneumoniae*	*P. aeruginosa*
	ESBL	ESBL	ESBL
**7a**	4	8	128
**7d**	16	16	32
**7e**	32	16	32
**9d**	128	–	128
**9e**	64	–	64
Ceftazidime	8	>128	32
Ciprofloxacin	>128	1	8

The evaluated compounds had a wide range of activity against MRSA, with the 5-*N*-isopropylamidinobenzimidazoles **8a**–**8e** being the most active (MIC = 8–32 µg/mL) (Table 3). **8a**–**8e** were also active against the MSSA strain, although to a lesser extent (MIC = 16–128 µg/ml). **8c** also displayed modest activity against VRE-*E*. *faecium.* Among other compounds, benzimidazole imidazoline **9c** had promising activity against the MRSA strain (MIC = 8 µg/ml). Against the antibiotic-resistant Gram-negative bacteria (Table 4), 5-amidinobenzimidazole **7a**, with the *N*-1-phenyl-1,2,3-triazole, proved to be the most potent, with IC_50_ values of 4 µg/ml for *E. coli*, and 8 µg/ml for *K. pneumoniae.* However, this compound was only marginally effective against *P. aeruginosa.* Compounds **7d** and **7e** prove to be active against *K. pneumoniae* (MIC = 16 µg/ml). Introduction of a methylene (**7d** and **9d**) and sulphide-bridge (**7e** and **9e**) between 1,2,3-triazole and the phenyl ring reduced the activity against the antibiotic resistant *E. coli* and *K. pneumoniae* clinical strains. **7d**, **7e**, and **9e** had slightly greater potency against *P. aeruginosa* compared with **7a**. Overall, the results indicated that the *o*-chlorophenyl hydrophobic unit, with *N*-isopropylamidine, as the hydrophilic unit, in **8c** contributed to anti-bacterial activity, particularly against the MRSA strain. Importantly, **7a** was the most potent of the compounds against ESBL-producing *E. coli*, with higher activity than the reference antibiotics ceftazidime and ciprofloxacin.

One of our aims was to determine if there was a relationship between the affinity of compounds towards ds-DNA/RNA and their antimicrobial activity. UV–Vis and CD spectroscopy, as well as thermal denaturation assays, showed that compounds **7b**, **9a**, and **9b**, which did not bind to *ct*DNA, had only marginal anti-microbial activities (MIC ≥128 µg/ml). Conversely, 5-amidinobenzimidazole **7a**, which showed the highest affinity to *ct*DNA, exhibited high potency against ESBL-producing *E. coli*, which is in agreement with previous findings^56^ ^,^ ^81^ ^,^ ^82^.

#### Screening of the anti-trypanosomal activity

Results on the *in vitro* testing against the bloodstream form *T. brucei* of the 5-amidinobezimidazoles **7a**–**7e**, **8a**–**8e**, and **9a**–**9e** with 1,4-disubstituted 1,2,3-triazole, and nifurtimox as reference drug, are summarised in Table 5. Similarly to anti-bacterial evaluations, we investigated how cationic moieties and aromatic substituents attached to the N-1 of the 1,2,3-triazole ring directly, or through the methylene and methylenesulphide spacer, influenced, anti-trypanosomal potencies.

**Table 5.**
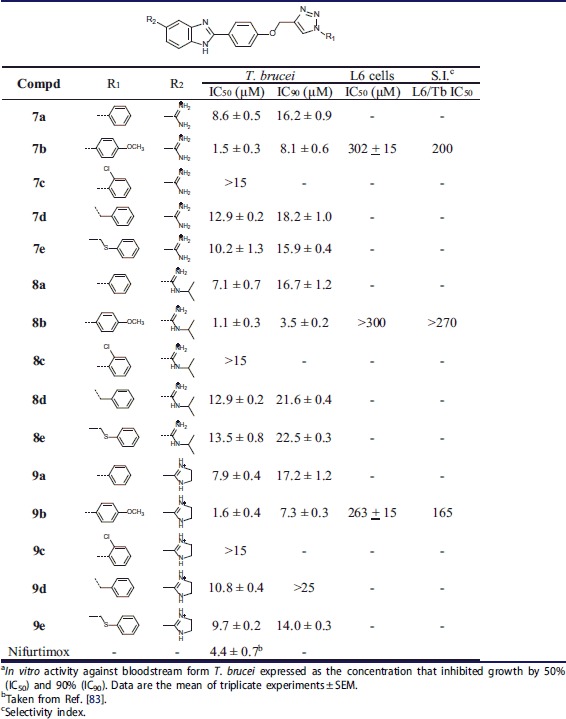
Anti-trypanosomal activity^a^ of compounds **7a**–**7e**, **8a**–**8e**, and **9a**–**9e** against *Trypanosoma brucei* strain.

Phenyl, *p*-methoxyphenyl, *o*-chlorophenyl, benzyl and (phenylthio)methyl substituents had a significant negative impact on IC_50_ values of anti-trypanosomal activity in the following order: *p*-OCH_3_ > Ph > PhSCH_2_ ≈ Bn > *o*-Cl. Except for **7c**–**9c**, all compounds were active against *T. brucei* with IC_50_ values ranging from 1.1 to 13.5 µM. Interestingly, the *o*-chlorophenyl substituent in **7c**–**9c** caused the loss of anti-trypanosomal activity (IC_50_ > 15 µM). The presence of the *p*-methoxyphenyl substituent in **7b**–**9b** led to enhanced anti-trypanosomal potency, with the 5-*N*-isopropylamidinobenzimidazole analogue **8b** being the most promising compound (IC_50_ = 1.1 µM, IC_90_ = 3.5 µM), which is 4-fold more potent than nifurtimox. UV–Vis titrations and thermal denaturation assays suggested that **7b**–**9b** have low affinity to *ct*DNA (Table 1) indicating that DNA is not the primary target for their anti-trypanosomal activity. Cytotoxicity assays against the rat myoblast cell line L6, revealed negligible activity, with three-figure selectivity index (Table 5).

## Conclusions

The 1,2,3-triazole-linked 5-amidinobenzimidazoles **7a**–**7e**, **8a**–**8e**, and **9a**–**9e** were synthesised by a Cu(I)catalysed 1,3-dipolar cycloaddition reaction applying microwave and ultrasound irradiation, with subsequent formation of a benzimidazole moiety by oxidative coupling of *o*-phenylenediamines with benzaldehydes. It was found that the **7c**–**9c**, **7d**–**9d**, and **7e**–**9e** sets of compounds non-covalently bound to ds-DNA/RNA. The small bathochromic shifts in UV–Vis titration spectra upon addition of *ct*DNA, modest thermal stabilisation effects, and strong positive ICD bands in CD titration experiments supported minor groove binding as the dominant binding mode of these compounds. Conversely, the appearance of negative ICD bands in CD titration experiments with polyA-polyU and polyC-polyG, and density of binding sites obtained from UV–Vis titrations, identified intercalation as the predominant binding mode.

Furthermore, SARs showed that the type of aromatic substituents at N-1 of 1,2,3-triazole had profound effects on anti-bacterial and anti-protozoal activities. Thus, results of anti-bacterial evaluations revealed that *o*-chlorophenyl-1,2,3-triazole and *N*-isopropylamidine moieties in **8c** had a considerable impact on inhibitory activity against resistant Gram-positive bacteria, particularly the MRSA strain. On the other hand, non-substituted amidine and phenyl rings in **7a** contributed to a strong inhibitory effect on an ESBL-producing *E. coli* strain, with the potency better than those of the reference antibiotics ceftazidime and ciprofloxacin Compounds **7 b**, **9a**, and **9 b** that showed extremely low affinity to *ct*DNA had also negligible anti-microbial activity (MIC ≥128 µg/ml). Contrary to this, the 5-*N*-isopropylamidinobenzimidazole series **8a**–**8e**, which had better binding affinity relative to other amidines, showed some selective activity (MIC = 8–32 µg/ml) against the MRSA strain. Notably, compound **7a** emerged as the most promising candidate because of its higher potency (MIC = 4 µg/ml) against ESBL-producing *E. coli.* It had the highest affinity among the tested compounds to *ct*DNA (Tables 1 and 2).

Results of anti-trypanosomal evaluations showed that the *o*-chlorophenyl group in **7c**–**9c** had a negative impact on activity, whereas the *p*-methoxyphenyl substituent in **7b**–**9b** enhanced activity, with **8b** (IC_50_ = 1.1 µM and IC_90_ = 3.5 µM) being more potent than nifurtimox. In contrast to the observed correlation between anti-microbial activity and DNA binding, the antiprotozoal effects of **8b** did not correlate with its DNA affinity. Further investigations will, therefore, be required to clarify the mechanism of anti-protozoal activity.

The promising anti-bacterial activity of compounds **7a** and **8c** and the anti-trypanosomal potency of compound **8b** suggest that further structural optimisation of the 1,2,3-triazole-linked 5-amidinobenzimidazole class could enhance the potential anti-HAT and anti-bacterial activity against resistant pathogenic microorganisms.

## Supplementary Material

Supplemental Material
