# Supplementary material for: Synthesis, anti-bacterial and anti-protozoal activities of amidinobenzimidazole derivatives and their interactions with DNA and RNA
Source: J Enzyme Inhib Med Chem. 2018 Aug 31;33(1):1323–34. doi: 10.1080/14756366.2018.1484733 (PMC6127852; doi:10.1080/14756366.2018.1484733)

Synthesis, anti-bacterial and anti-protozoal activities of amidinobenzimidazole derivatives and their interactions with DNA and RNA

Andrea Bistrović[a], Luka Krstulović[b], Ivana Stolić[b], Domagoj Drenjančević[c,d], Jasminka Talapko[d], Martin C. Taylor[e], John M. Kelly[e], Miroslav Bajić[b] and Silvana Raić-Malić[a]*

[a]Department of Organic Chemistry, Faculty of Chemical Engineering and Technology, University of Zagreb, Marulićev trg 20, HR-10000 Zagreb, Croatia; [b]Department of Chemistry and Biochemistry, Faculty of Veterinary Medicine, University of Zagreb, Heinzelova 55, 10000 Zagreb, Croatia; [c]Department of Transfusion Medicine, Osijek University Hospital, Josipa Huttlera 4, HR-31000 Osijek, Croatia ;[d]Faculty of Medicine, Department of Microbiology and Parasitology,University of Osijek, Cara Hadrijana 10/E, HR-31000 Osijek, Croatia; [e]Department of Pathogen Molecular Biology, London School of Hygiene and Tropical Medicine, Keppel Street, London, WC1E 7HT, UK

*Corresponding Author:

Tel.: 00385-1-4597-213. Fax.: 00385-1-4597-224. E-mail: sraic@fkit.hr;

Content	Pages
1. UV/VIS specrta of novel compounds	S2–S6
2. CD titrations of novel compounds	S7–S10
3. Antimicrobial activities of novel compounds against Gram-positive and Gram-negative bacteria.	S11
3. ^{1}H and ^{13}C NMR spectra of novel compounds	S12–S22

1. Spectroscopic characterization of compounds

Due to the low solubility of investigate compounds in pure water, a stock solutions were prepared in DMSO, $c = 1 \times 10^{-3}$ mol dm^{-3}. Stock solutions have been characterized by UV-Vis spectroscopy. DMSO solutions of all studied compounds were shown to be stable over longer period at 4−8 °C. Table 1 gives values for the molar absorption coefficients of compounds in water-DMSO (≥ 1 %) mixture.

Table S1. Molar absorption coefficients of investigated compounds

	λ_{max} / nm	$\varepsilon \cdot 10^3$ / dm^{3}mol^{-1}cm^{-1}		λ_{max} / nm	$\varepsilon \cdot 10^3$ / dm^{3}mol^{-1}cm^{-1}		λ_{max} / nm	$\varepsilon \cdot 10^3$ / dm^{3}mol^{-1}cm^{-1}
**7a**	257<br>315	30.7<br>31.4	**8a**	255<br>313	22.4<br>23.1	**9a**	259<br>313	24.9<br>22.4
**7b**	259<br>313	29.6<br>27.0	**8b**	256<br>312	28.1<br>27.5	**9b**	261<br>321	30.5<br>25.2
**7c**	256<br>313	26.9<br>28.7	**8c**	255<br>313	24.3<br>30.0	**9c**	258<br>317	32.5<br>35.9
**7d**	257<br>313	27.5<br>35.1	**8d**	255<br>313	22.1<br>26.9	**9d**	259<br>317	23.3<br>26.9
**7e**	257<br>314	31.6<br>37.7	**8e**	254<br>312	20.6<br>24.3	**9e**	259<br>319	34.5<br>35.3

Fig. S1. UV/VIS spectra

	***ct*DNA**	**polyA-polyU**	**polyC-polyG**
**7a**	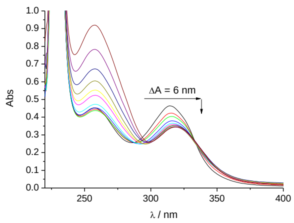	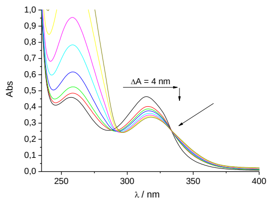	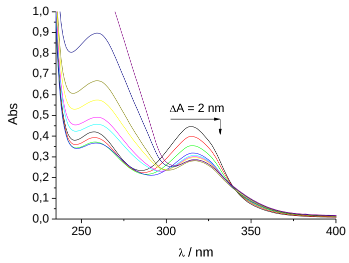

8a

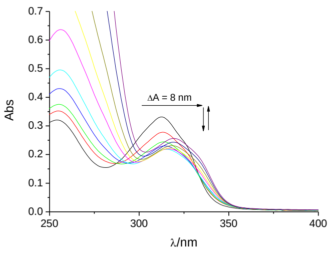
Abs
λ/nm
ΔA = 8 nm
0.0
0.1
0.2
0.3
0.4
0.5
0.6
0.7
250
300
350
400

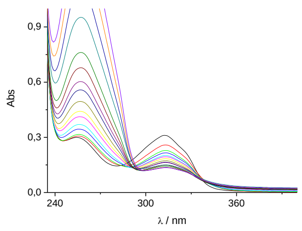
Abs
λ / nm
0,0
0,3
0,6
0,9
240
300
360

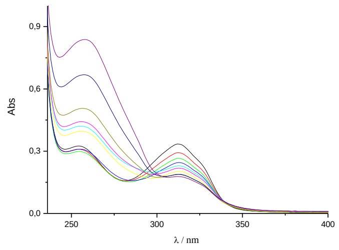
Abs
λ / nm
0,0
0,3
0,6
0,9
250
300
350
400

9a

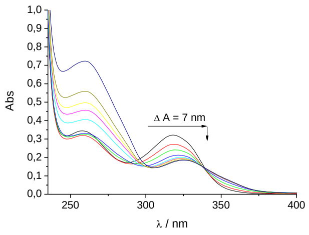
Abs
λ / nm
Δ A = 7 nm
0,0
0,1
0,2
0,3
0,4
0,5
0,6
0,7
0,8
0,9
1,0
250
300
350
400

7b

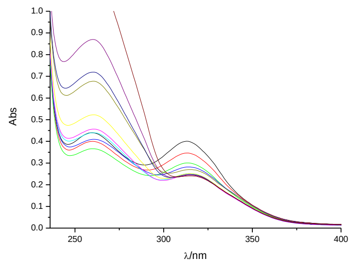
Abs
λ/nm
0.0
0.1
0.2
0.3
0.4
0.5
0.6
0.7
0.8
0.9
1.0
250
300
350
400

8b

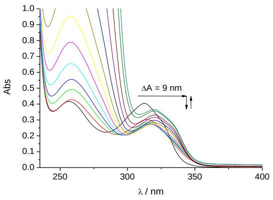
Abs
λ / nm
ΔA = 9 nm
0.0
0.1
0.2
0.3
0.4
0.5
0.6
0.7
0.8
0.9
1.0
250
300
350
400

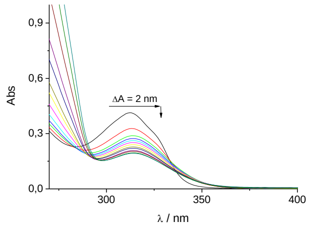
Abs
λ / nm
ΔA = 2 nm
0,0
0,3
0,6
0,9
300
350
400

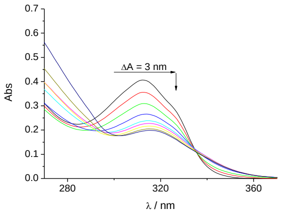
Abs
λ / nm
ΔA = 3 nm
0.0
0.1
0.2
0.3
0.4
0.5
0.6
0.7
280
320
360

9b

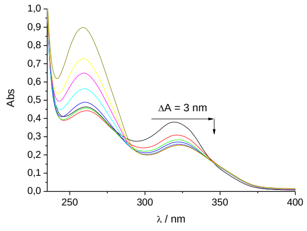
Abs
λ / nm
ΔA = 3 nm
0,0
0,1
0,2
0,3
0,4
0,5
0,6
0,7
0,8
0,9
1,0
250
300
350
400

	*ct*DNA	polyA-polyU	polyC-polyG
**7c**	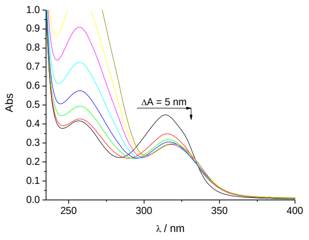	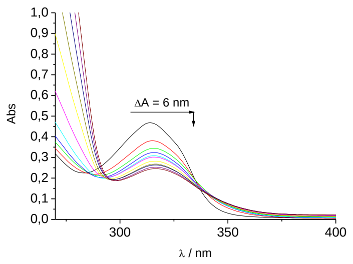	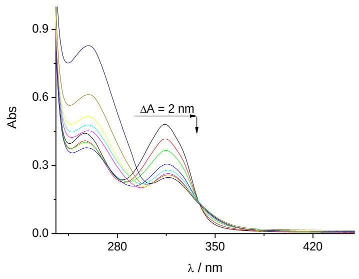
**8c**	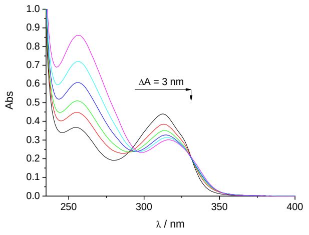	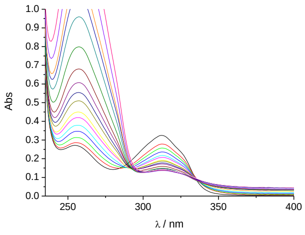	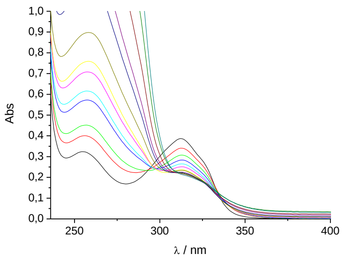
**9c**	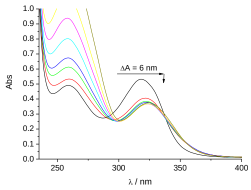	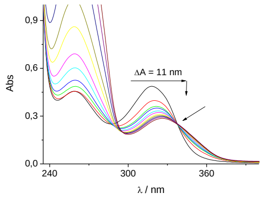	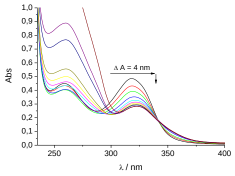

	*ct*DNA	polyA-polyU	polyC-polyG
**7d**	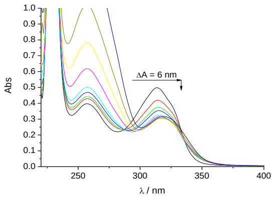	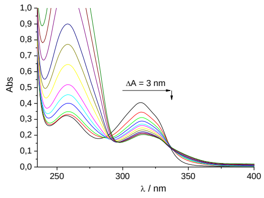	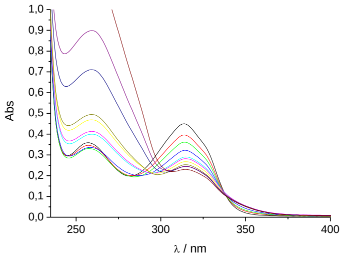

**8d**	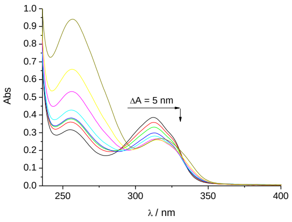	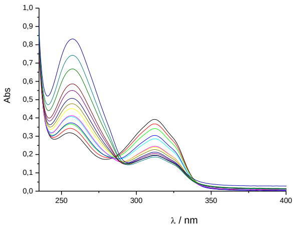	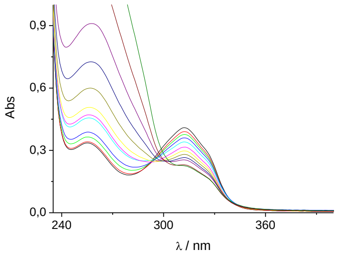
**9d**	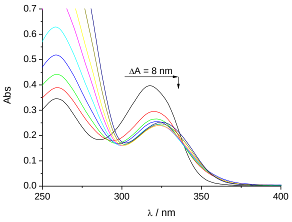	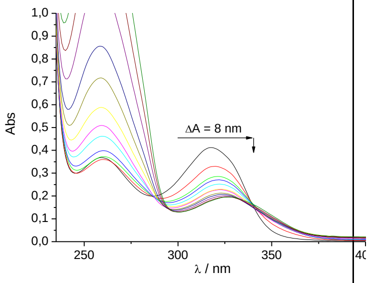	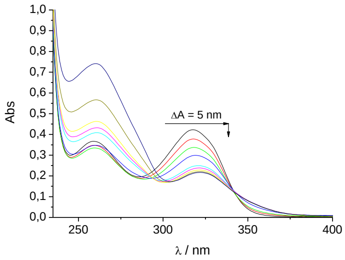

	***ct*DNA**	**polyA-polyU**	**polyC-polyG**
**7e**	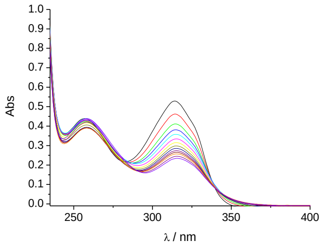	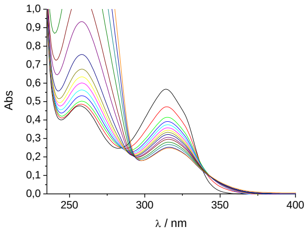	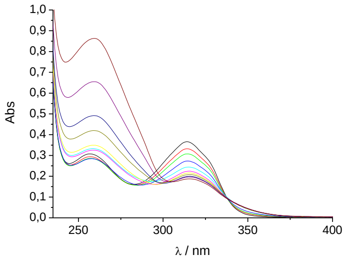
**8e**	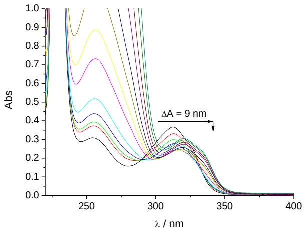	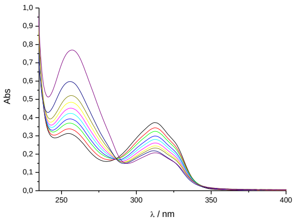	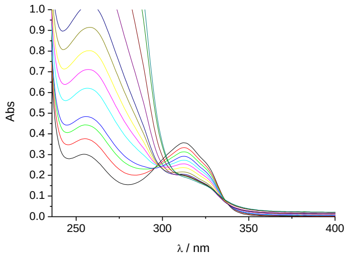

**9e**	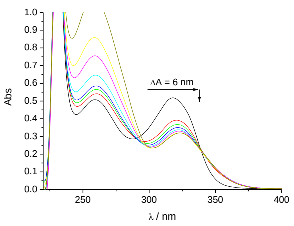	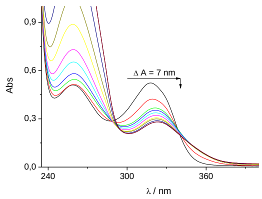	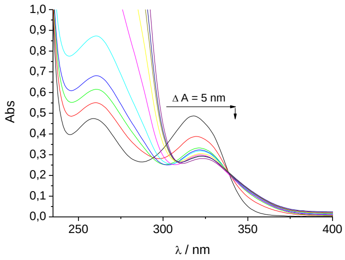

Fig. S2. CD titrations

<table>
<tr><th></th><th>ctDNA</th><th>polyA-polyU</th><th>polyC-polyG</th></tr>
<tr><td>7a</td><td></td><td>
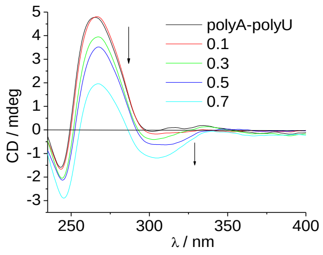

</td><td>
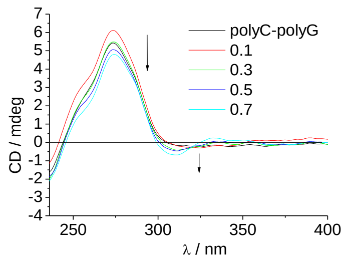

</td></tr>
<tr><td>8a</td><td>
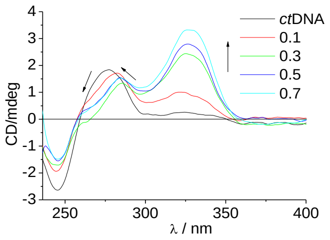

</td><td>
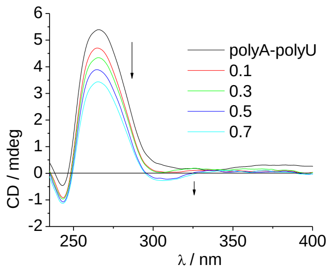

</td><td>
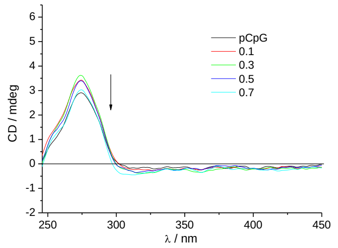

</td></tr>
<tr><td>9a</td><td></td><td></td><td>
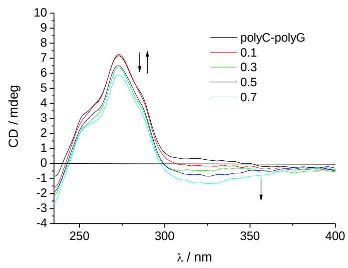

</td></tr>
<tr><td>7b</td><td></td><td></td><td>
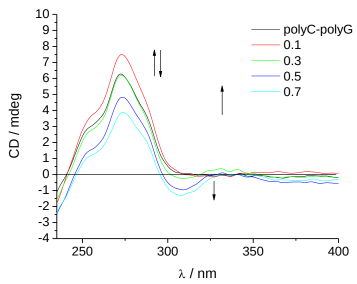

</td></tr>
</table>

8b

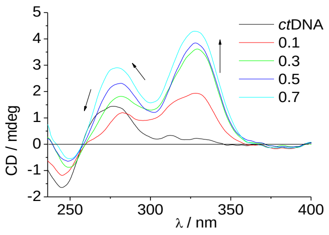
ctDNA
0.1
0.3
0.5
0.7
CD / mdeg
λ / nm

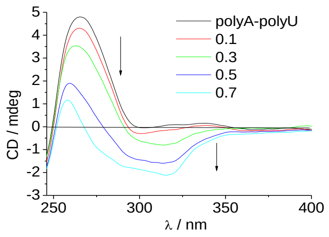
polyA-polyU
0.1
0.3
0.5
0.7
CD / mdeg
λ / nm

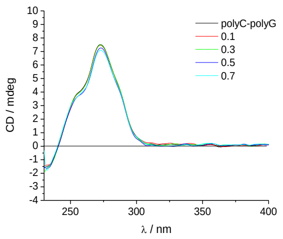
polyC-polyG
0.1
0.3
0.5
0.7
CD / mdeg
λ / nm

9b

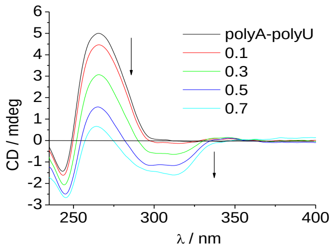
polyA-polyU
0.1
0.3
0.5
0.7
CD / mdeg
λ / nm

7c

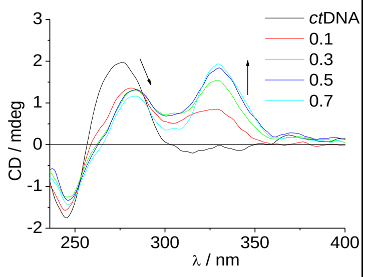
ctDNA
0.1
0.3
0.5
0.7
CD / mdeg
λ / nm

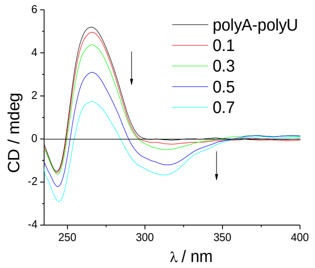
polyA-polyU
0.1
0.3
0.5
0.7
CD / mdeg
λ / nm

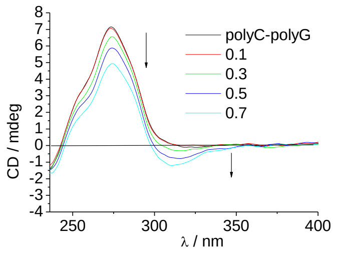
polyC-polyG
0.1
0.3
0.5
0.7
CD / mdeg
λ / nm

8c

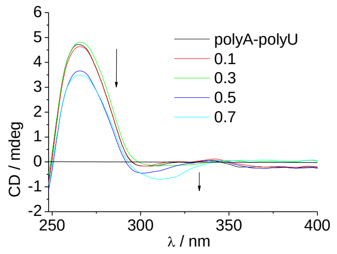
polyA-polyU
0.1
0.3
0.5
0.7
CD / mdeg
λ / nm

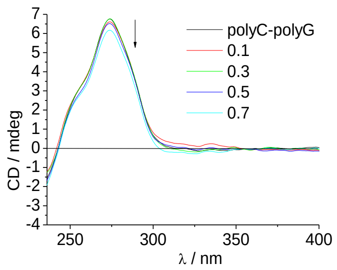
polyC-polyG
0.1
0.3
0.5
0.7
CD / mdeg
λ / nm

9c

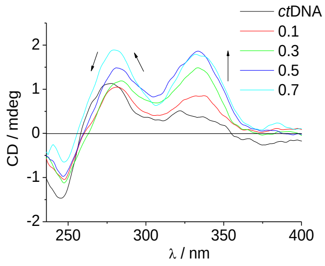
ctDNA
0.1
0.3
0.5
0.7
CD / mdeg
λ / nm

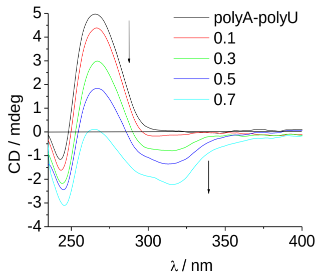
polyA-polyU
0.1
0.3
0.5
0.7
CD / mdeg
λ / nm

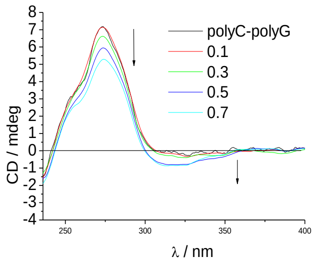
polyC-polyG
0.1
0.3
0.5
0.7
CD / mdeg
λ / nm

7d

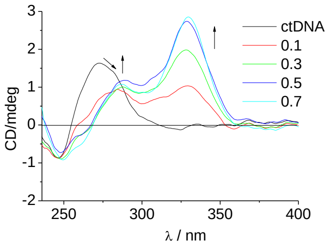
ctDNA
0.1
0.3
0.5
0.7
CD/mdeg
λ / nm

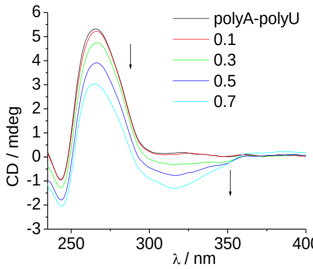
polyA-polyU
0.1
0.3
0.5
0.7
CD / mdeg
λ / nm

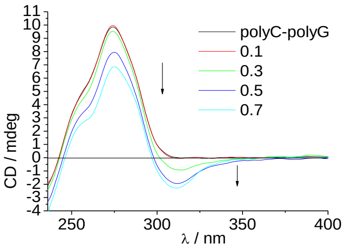
polyC-polyG
0.1
0.3
0.5
0.7
CD / mdeg
λ / nm

8d

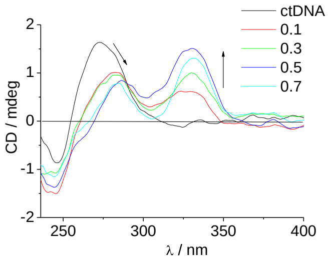
ctDNA
0.1
0.3
0.5
0.7
CD / mdeg
λ / nm

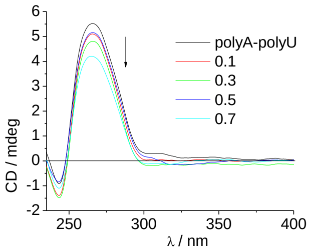
polyA-polyU
0.1
0.3
0.5
0.7
CD / mdeg
λ / nm

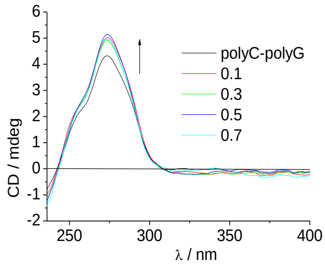
polyC-polyG
0.1
0.3
0.5
0.7
CD / mdeg
λ / nm

9d

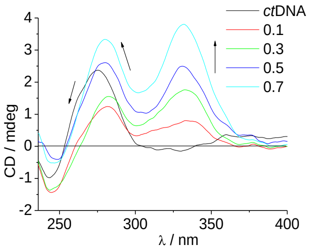
ctDNA
0.1
0.3
0.5
0.7
CD / mdeg
λ / nm

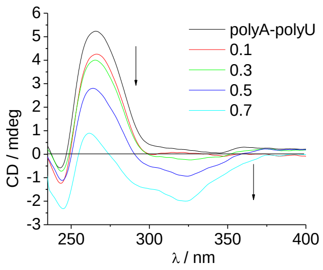
polyA-polyU
0.1
0.3
0.5
0.7
CD / mdeg
λ / nm

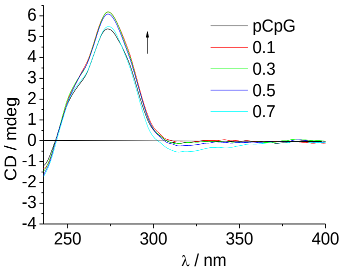
pCpG
0.1
0.3
0.5
0.7
CD / mdeg
λ / nm

7e

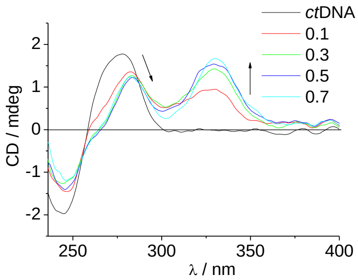
ctDNA
0.1
0.3
0.5
0.7
CD / mdeg
λ / nm

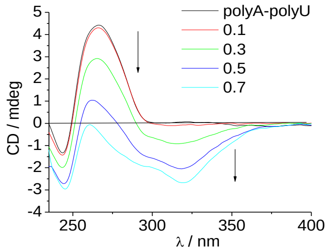
polyA-polyU
0.1
0.3
0.5
0.7
CD / mdeg
λ / nm

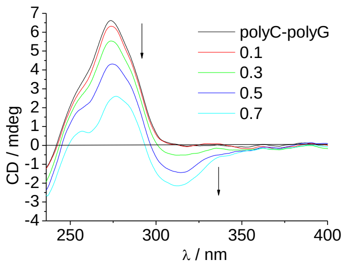
polyC-polyG
0.1
0.3
0.5
0.7
CD / mdeg
λ / nm

8e

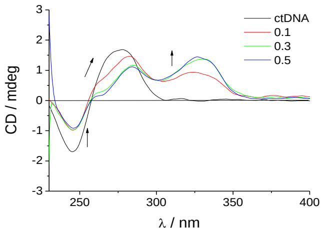
CD / mdeg
λ / nm
ctDNA
0.1
0.3
0.5

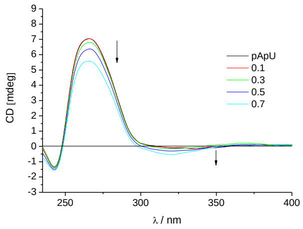
CD [mdeg]
λ / nm
pApU
0.1
0.3
0.5
0.7

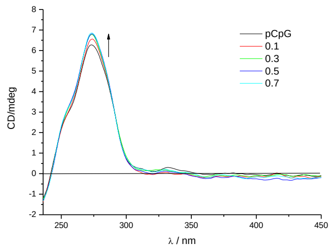
CD/mdeg
λ / nm
pCpG
0.1
0.3
0.5
0.7

9e

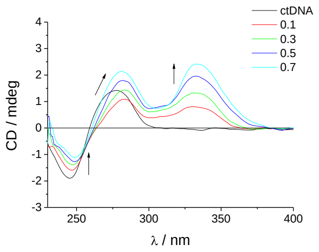
CD / mdeg
λ / nm
ctDNA
0.1
0.3
0.5
0.7

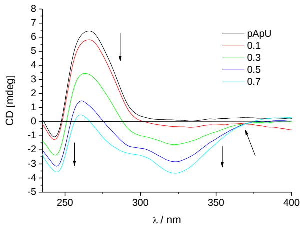
CD [mdeg]
λ / nm
pApU
0.1
0.3
0.5
0.7

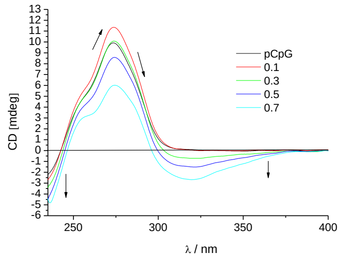
CD [mdeg]
λ / nm
pCpG
0.1
0.3
0.5
0.7

3. Antimicrobial activities

Table S2

Antimicrobial activities of compounds **7a**–**7e**, **8a**–**8e** and **9a**–**9e** against Gram-positive and Gram-negative bacteria.

**Compd**	**MIC (µg/mL)**					
	S. aureus ATCC 25923	*E. faecalis* ATCC 29212	*E. coli* ATCC 25925	*K. pneumonia* ATCC 700803	*P. aeruginosa* ATCC 27853	*A. baumannii* ATCC 19606
**7a**	32	64	32	128	128	>256
**7b**	>256	128	>256	>256	>256	>256
**7c**	64	64	256	>256	>256	>256
**7d**	32	64	32	64	32	>256
**7e**	32	128	32	128	64	>256
**8a**	128	256	256	>256	>256	>256
**8b**	128	64	256	>256	>256	>256
**8c**	32	64	256	>256	>256	>256
**8d**	64	128	>256	>256	>256	>256
**8e**	64	256	256	>256	>256	>256
**9a**	128	128	256	>256	>256	>256
**9b**	128	256	256	>256	>256	>256
**9c**	128	32	256	>256	>256	>256
**9d**	64	128	128	>256	128	>256
**9e**	32	128	64	>256	128	>256
**Ceftazidime**	-	-	0.5	-	4	-
**Ciprofloxacin**	-	-	0.004	0.06	0.5	0.06
**Ampicilin**	1	2	-	-	-	-
**Gentamicin**	0.125	8	-	-	-	-

Fig. S3 a) ^{1}H NMR and b) ^{13}C NMR of compd. **3c**

a)

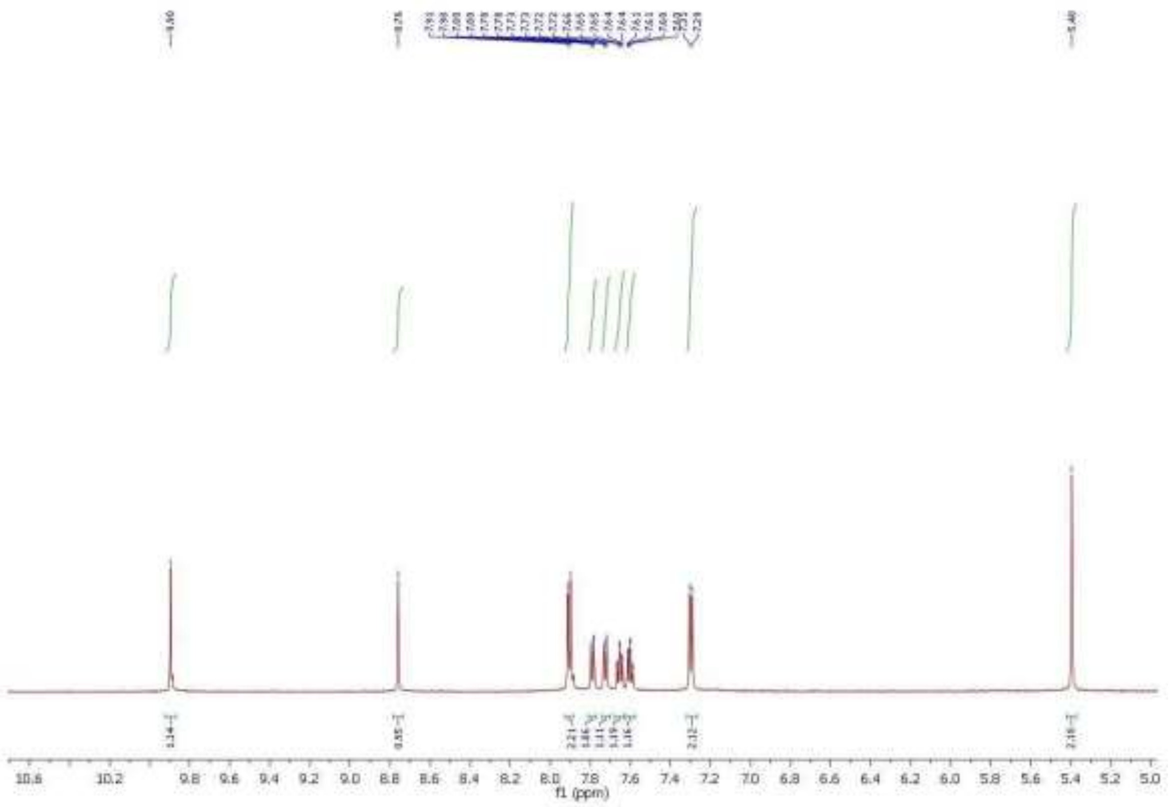

b)

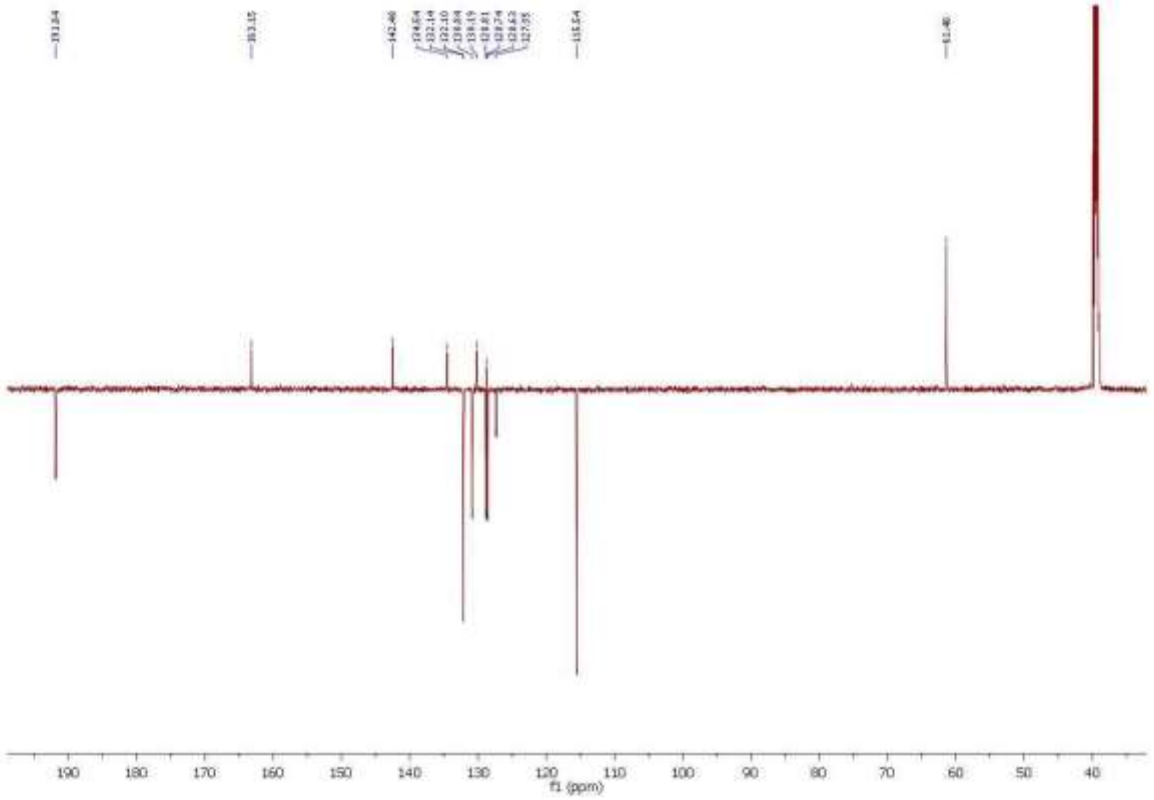

Fig. S4 a) ^{1}H NMR and b) ^{13}C NMR of compd. **3e**

a)

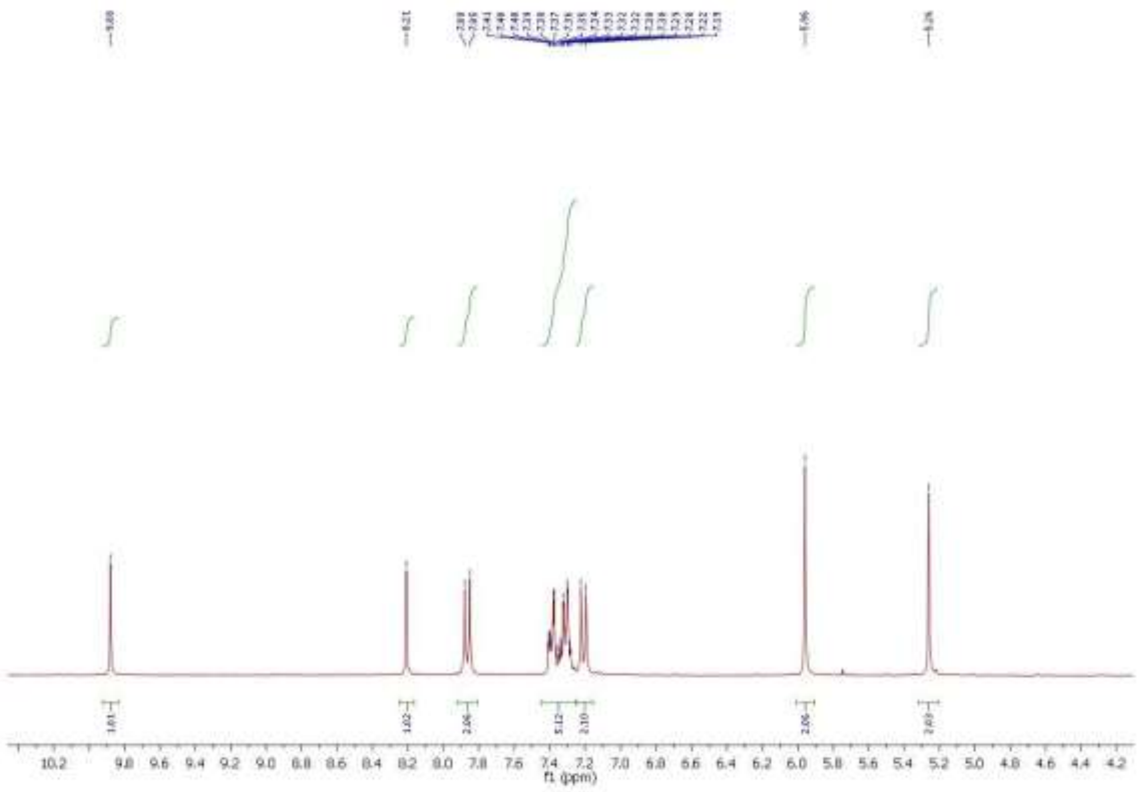

b)

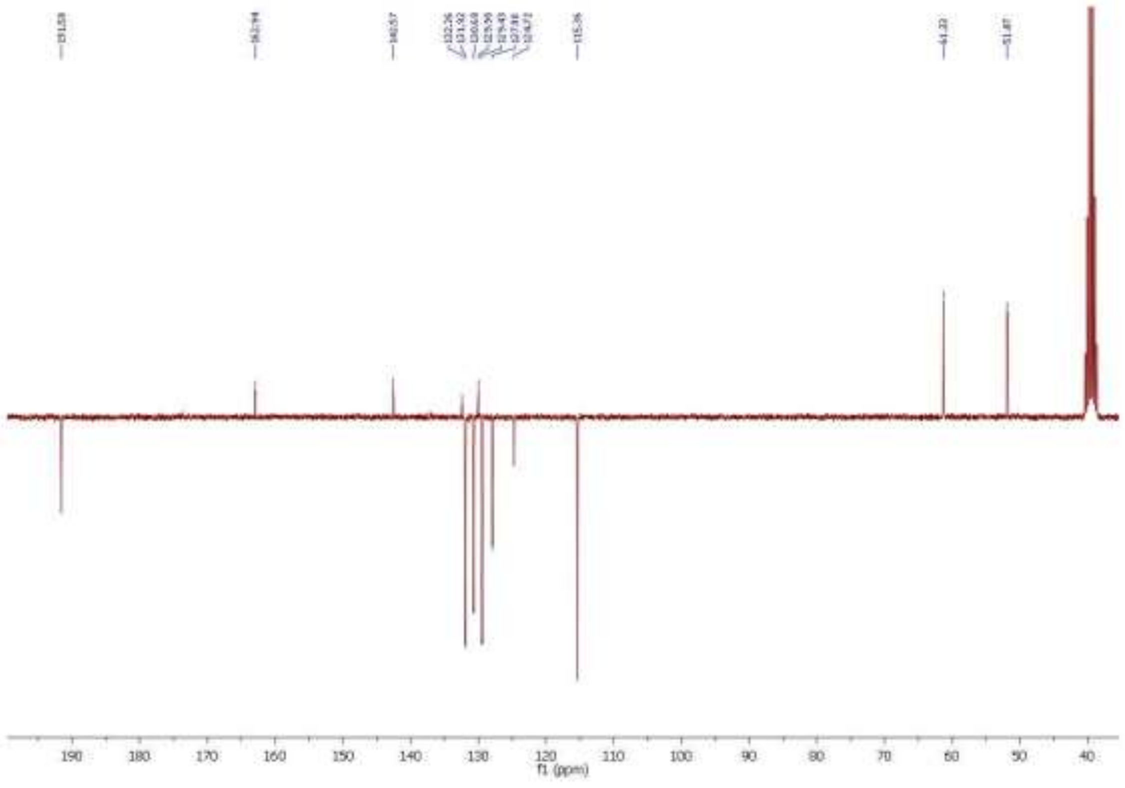

Fig. S5 a) ^{1}H NMR and b) ^{13}C NMR of compd. **7b**

a)

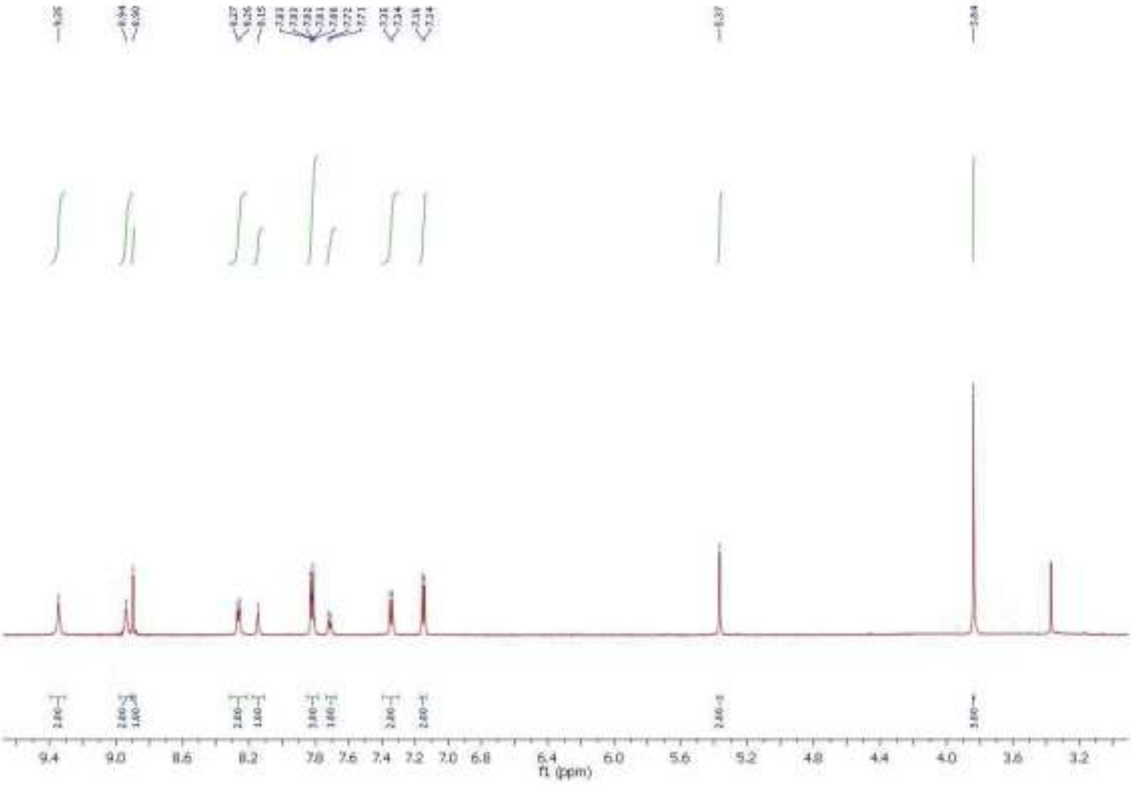

b)

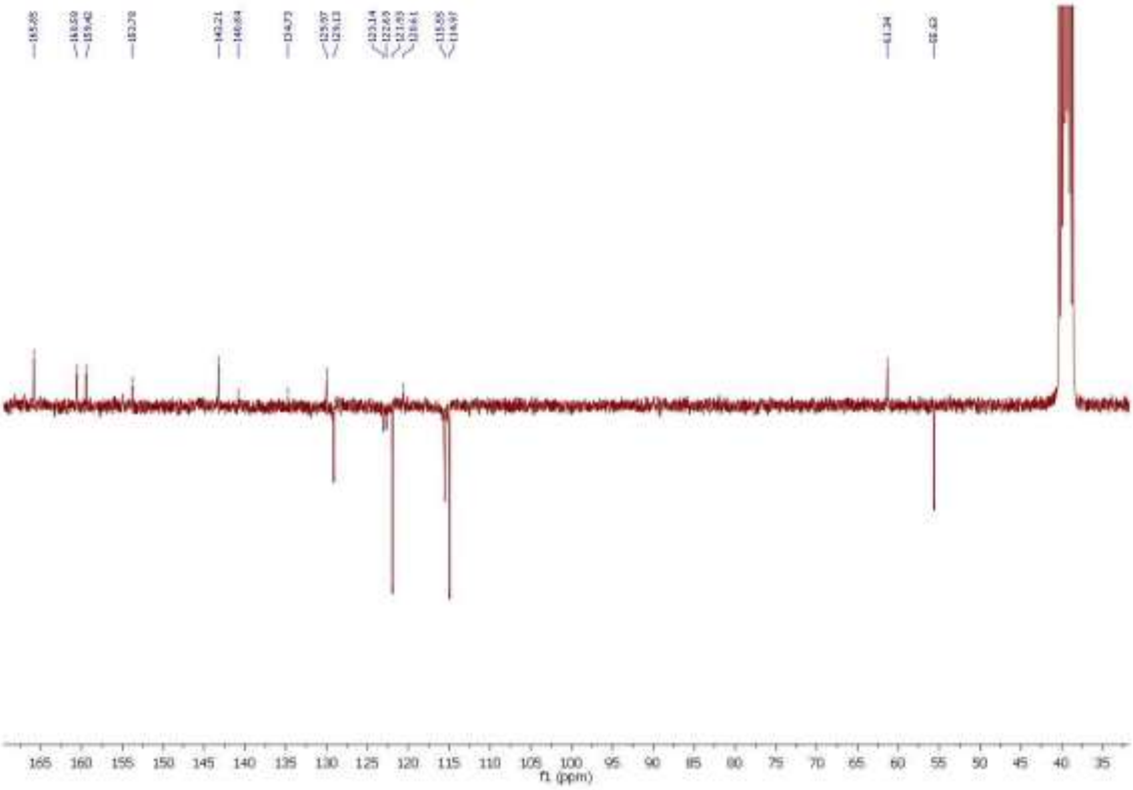

Fig. S6 a) ^{1}H NMR and b) ^{13}C NMR of compd. **7c**

a)

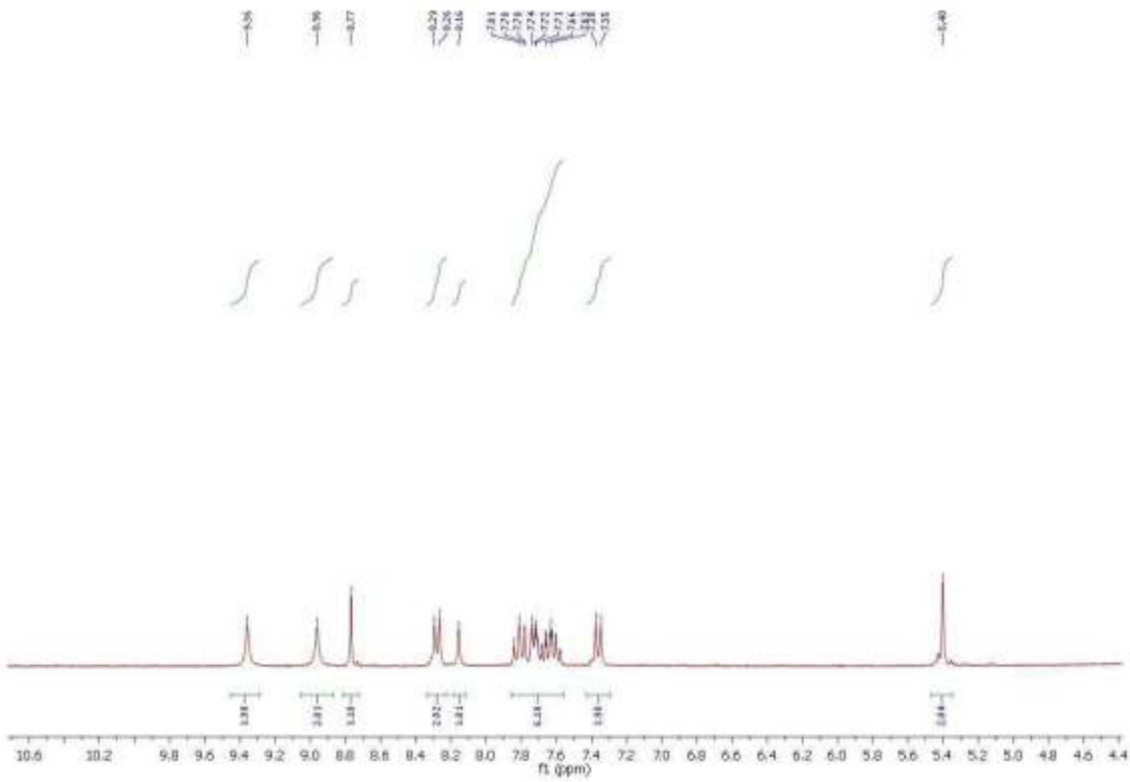

b)

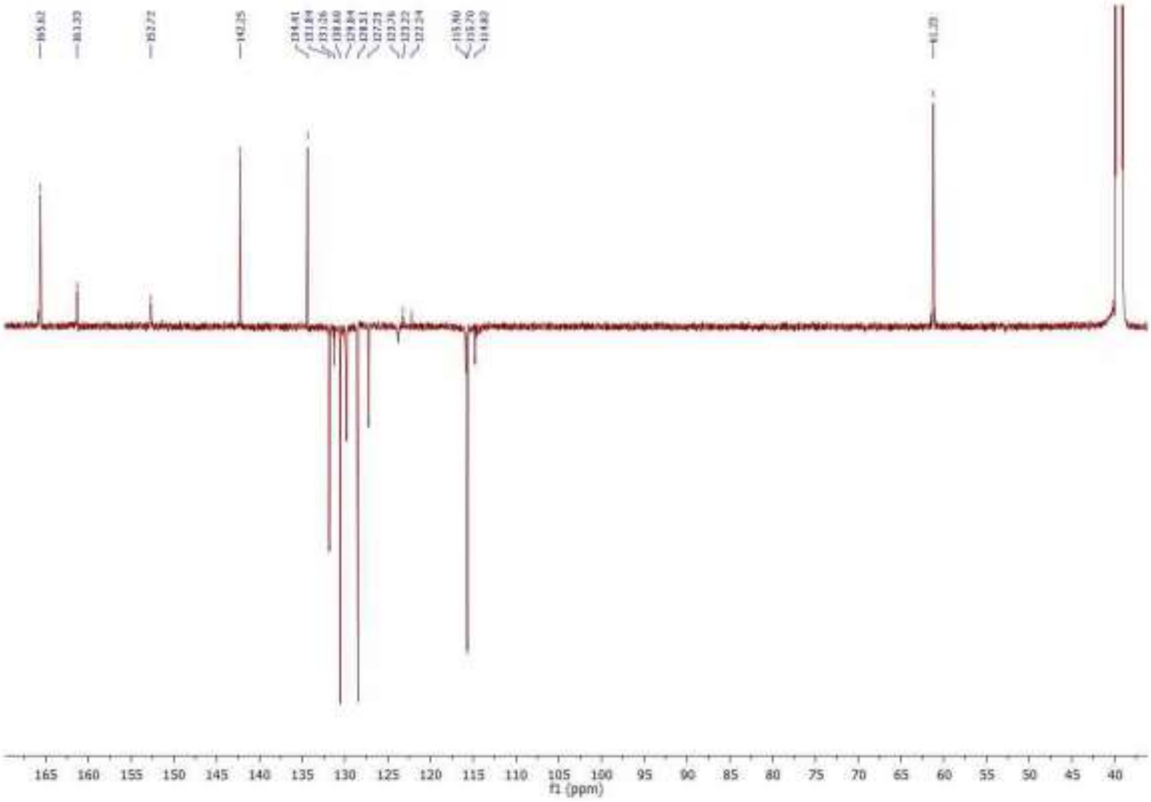

Fig. S7 a) ^{1}H NMR and b) ^{13}C NMR of compd. **7e**

a)

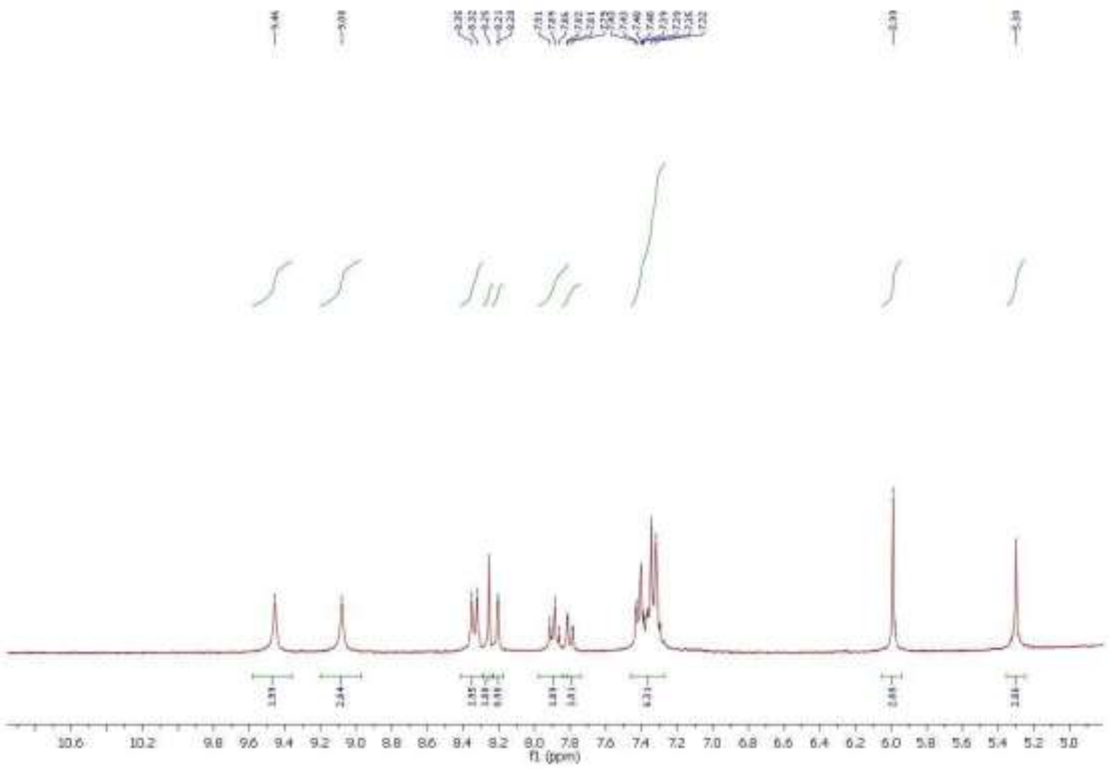

b)

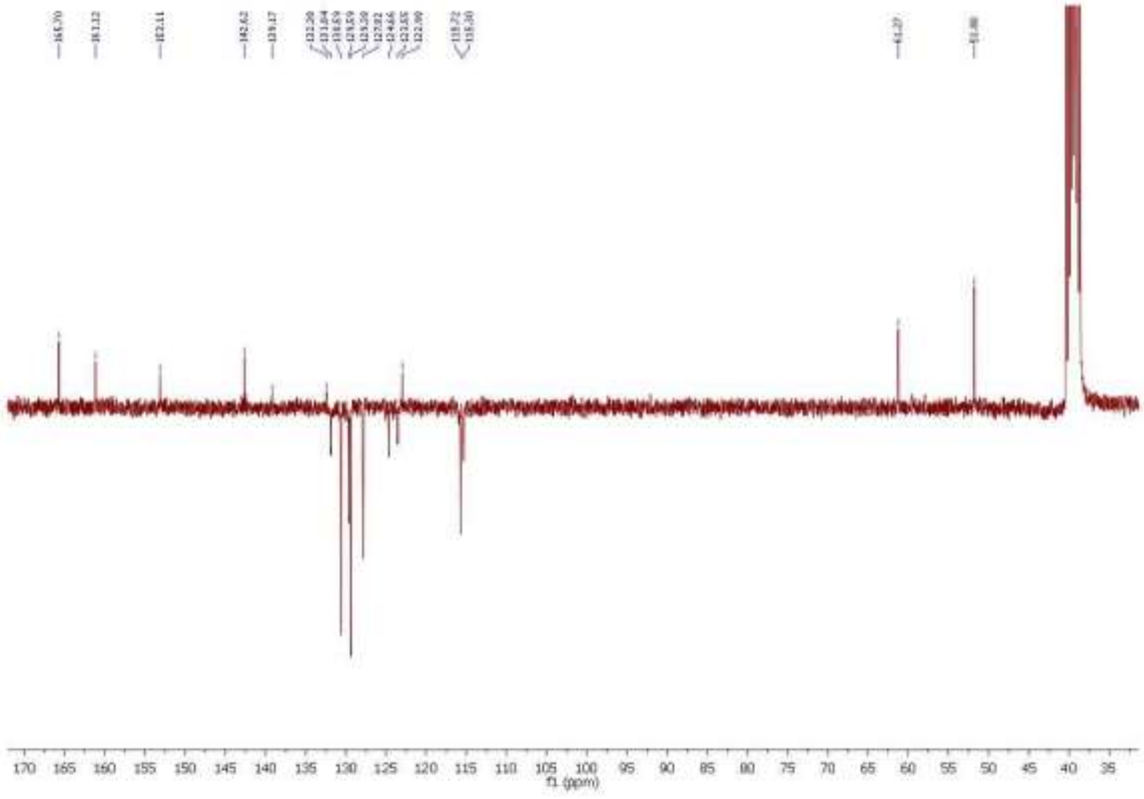

Fig. S8 a) ^{1}H NMR and b) ^{13}C NMR of compd. **8b**

a)

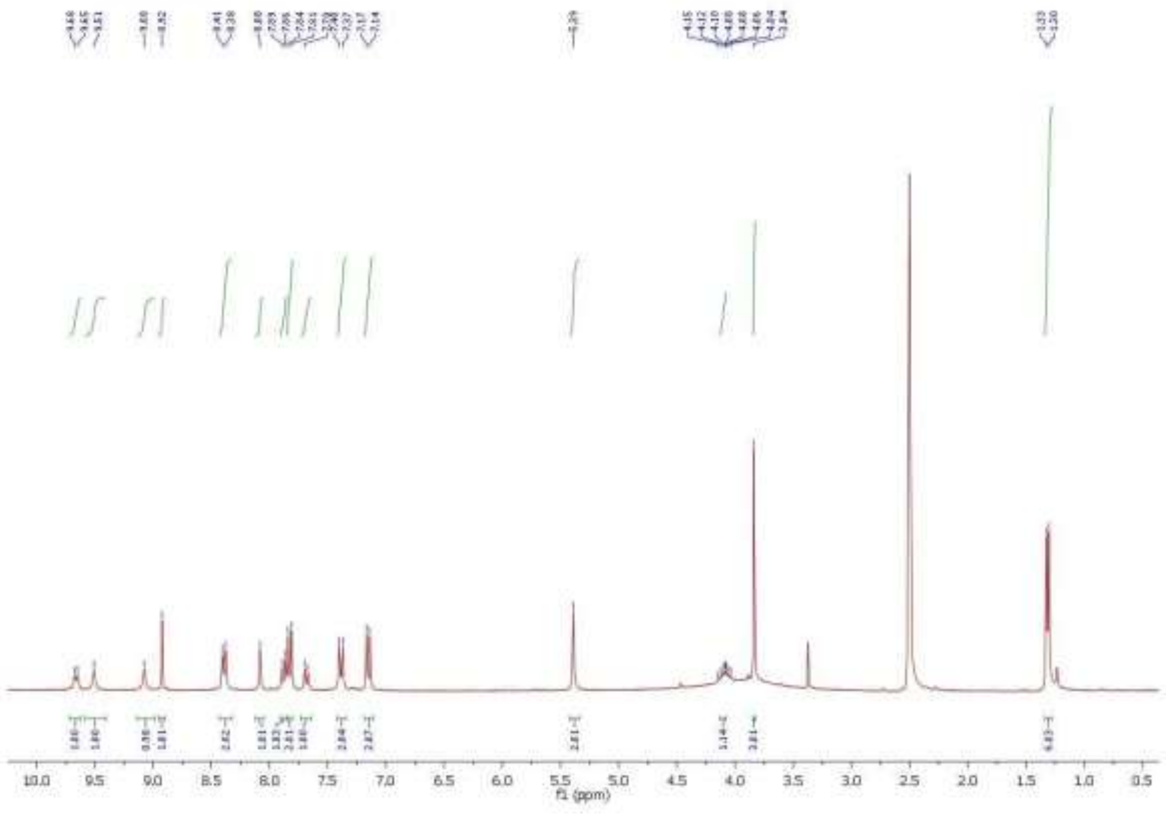

b)

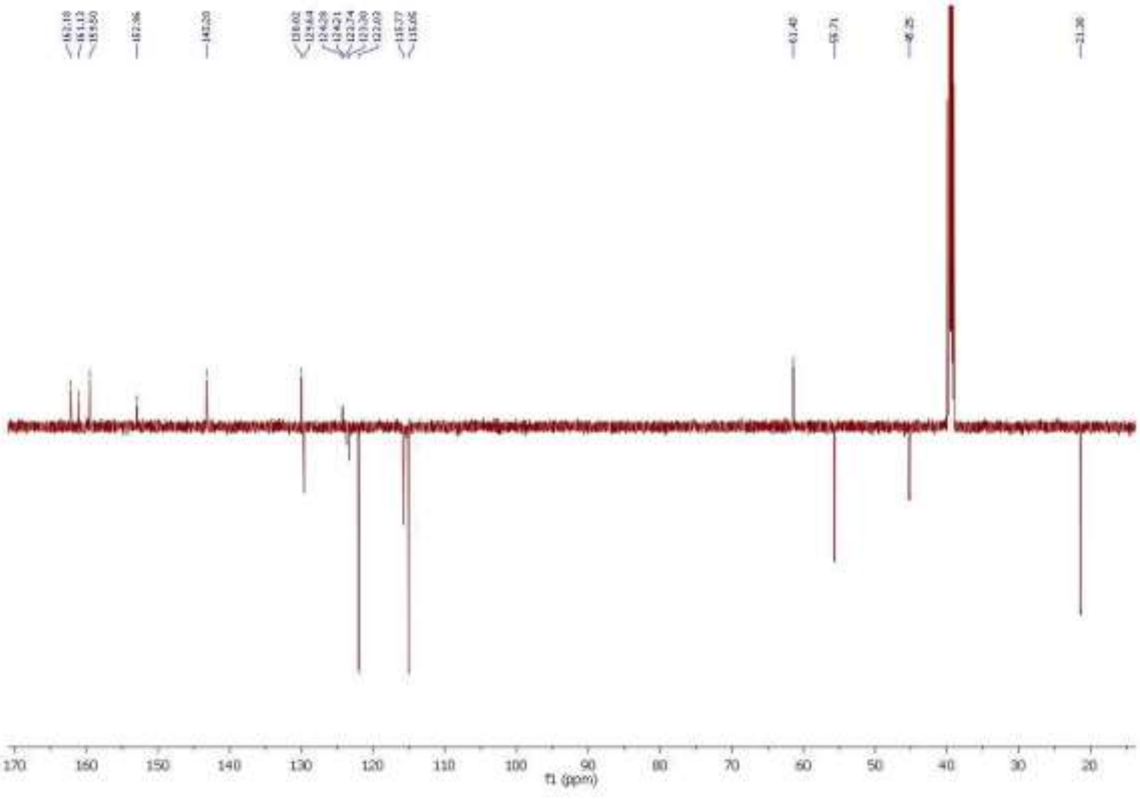

Fig. S9 a) ^{1}H NMR and b) ^{13}C NMR of compd. **8c**

a)

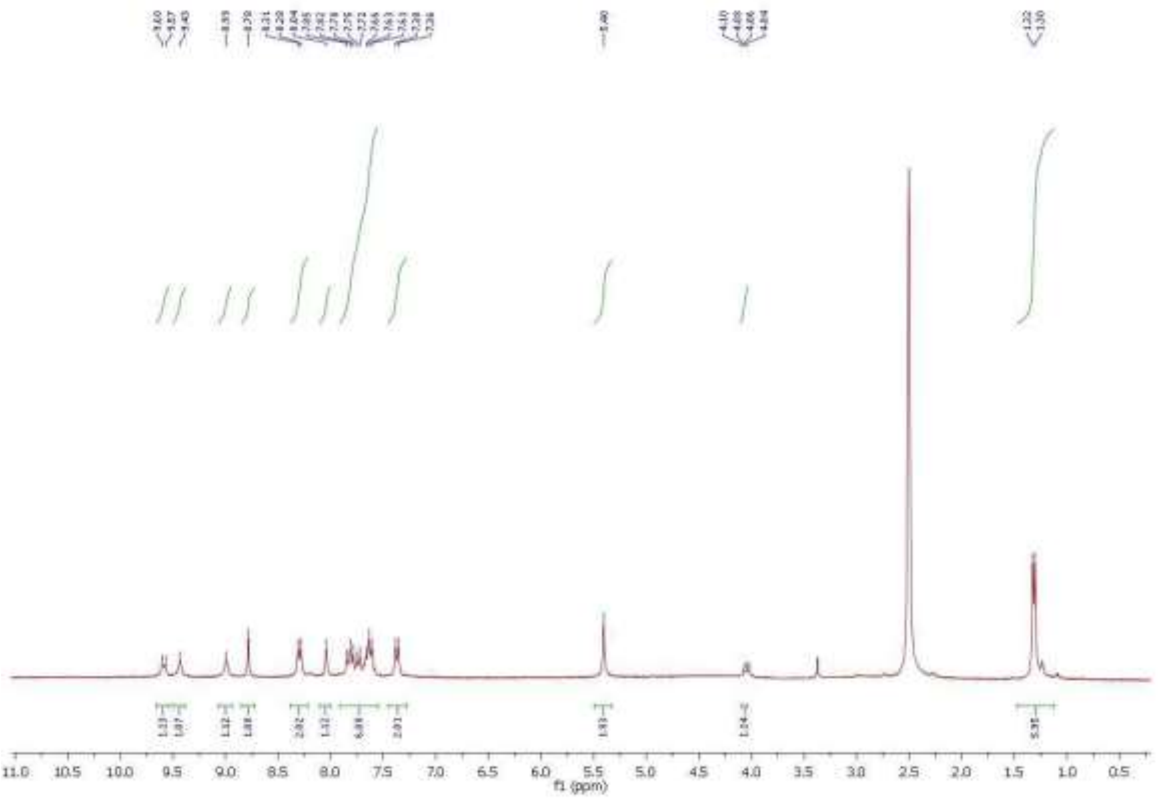

b)

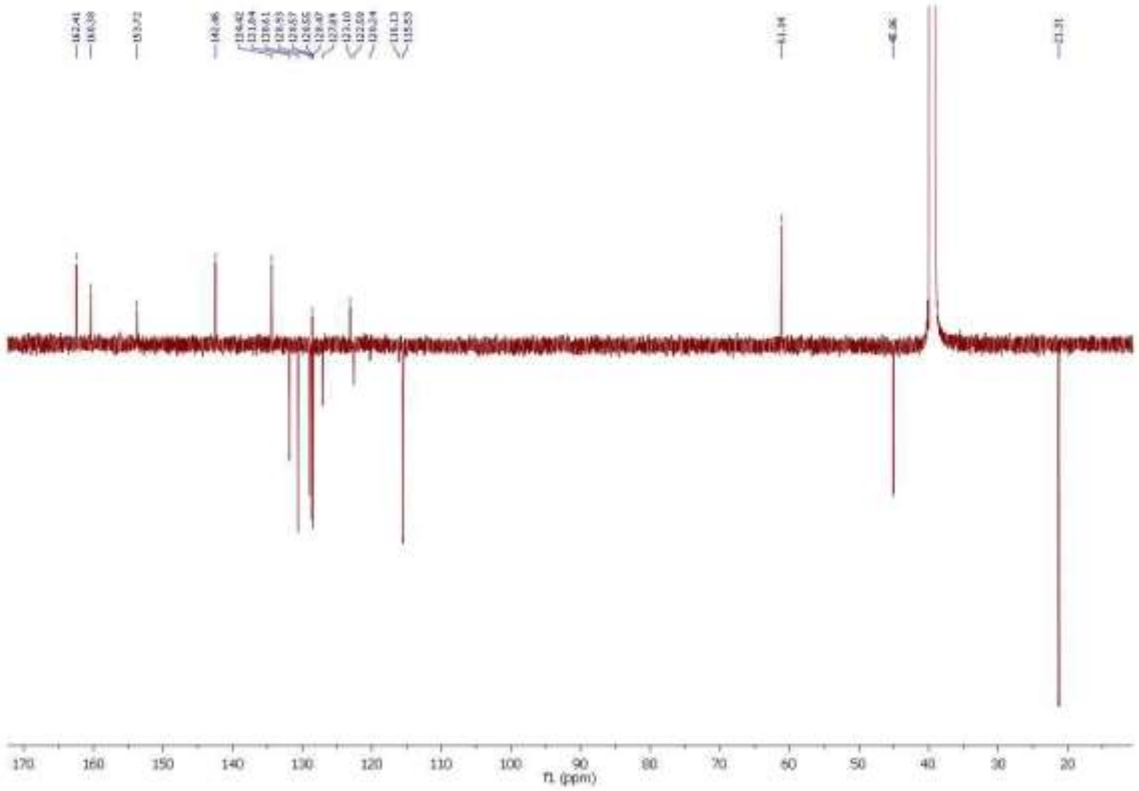

Fig. S10 a) ^{1}H NMR and b) ^{13}C NMR of compd. **8e**

a)

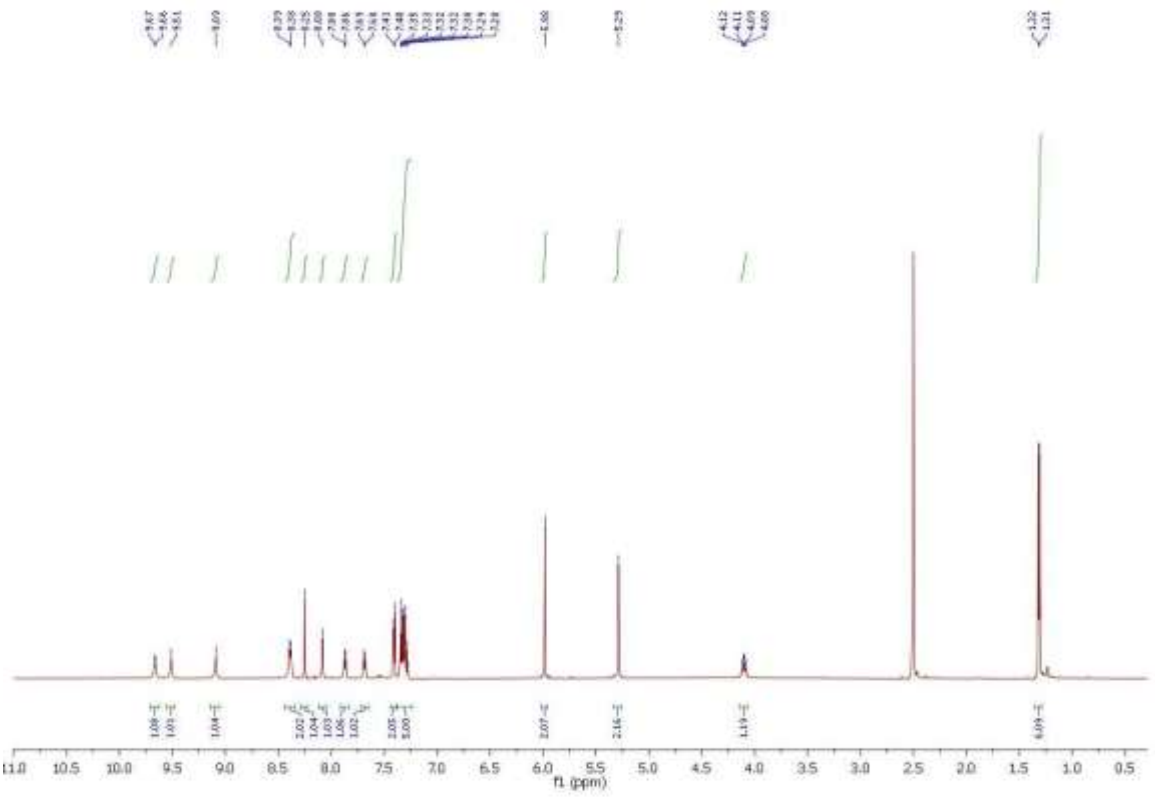

b)

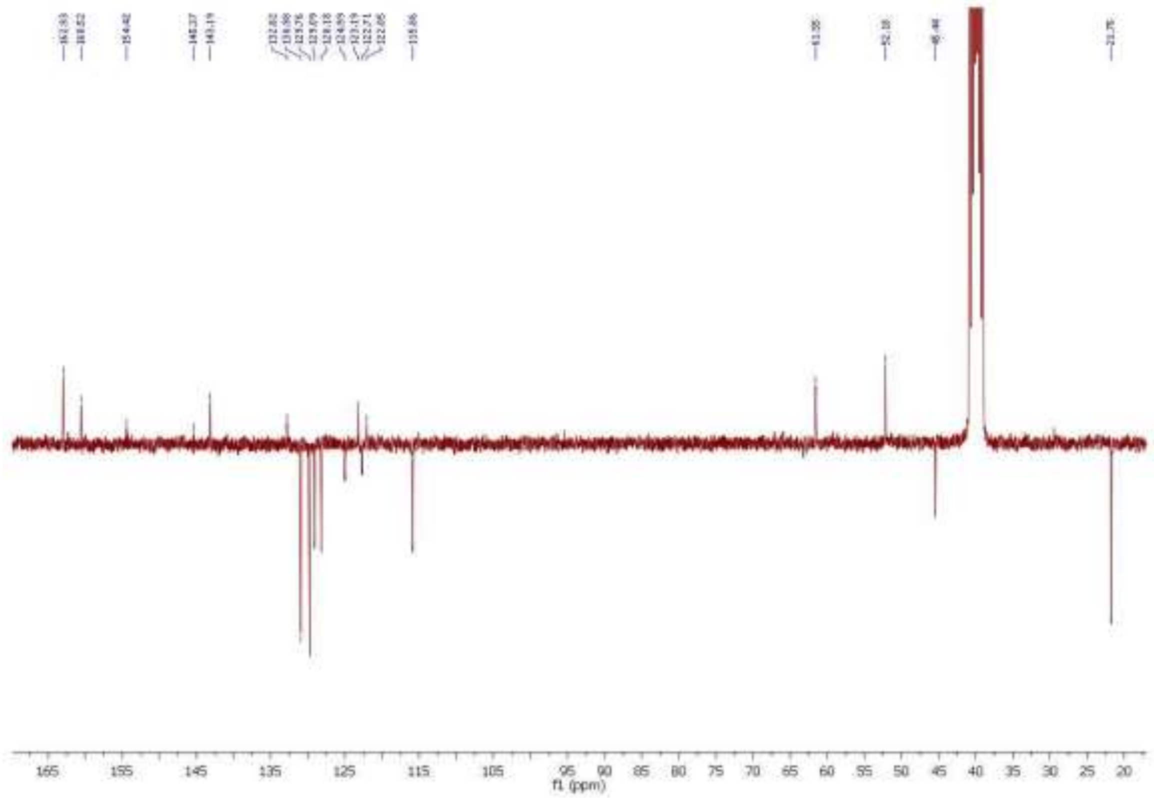

Fig. S11 a) ^{1}H NMR and b) ^{13}C NMR of compd. **9b**

a)

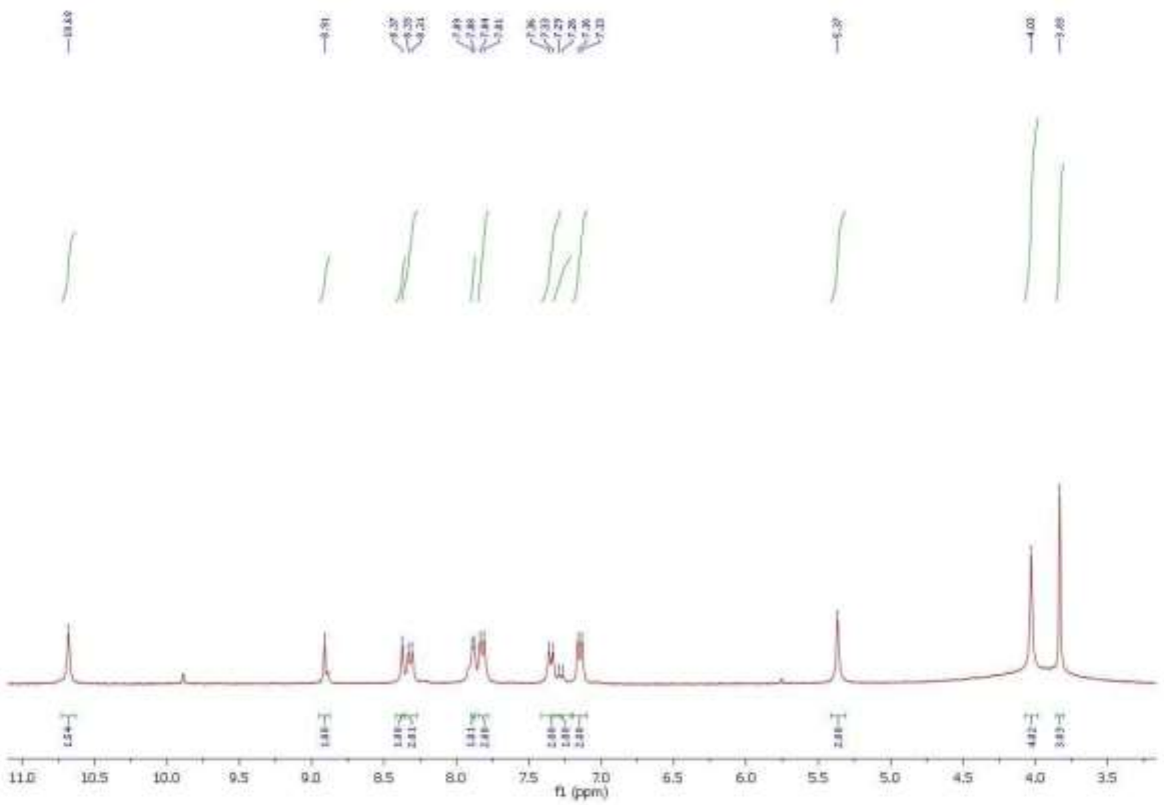

b)

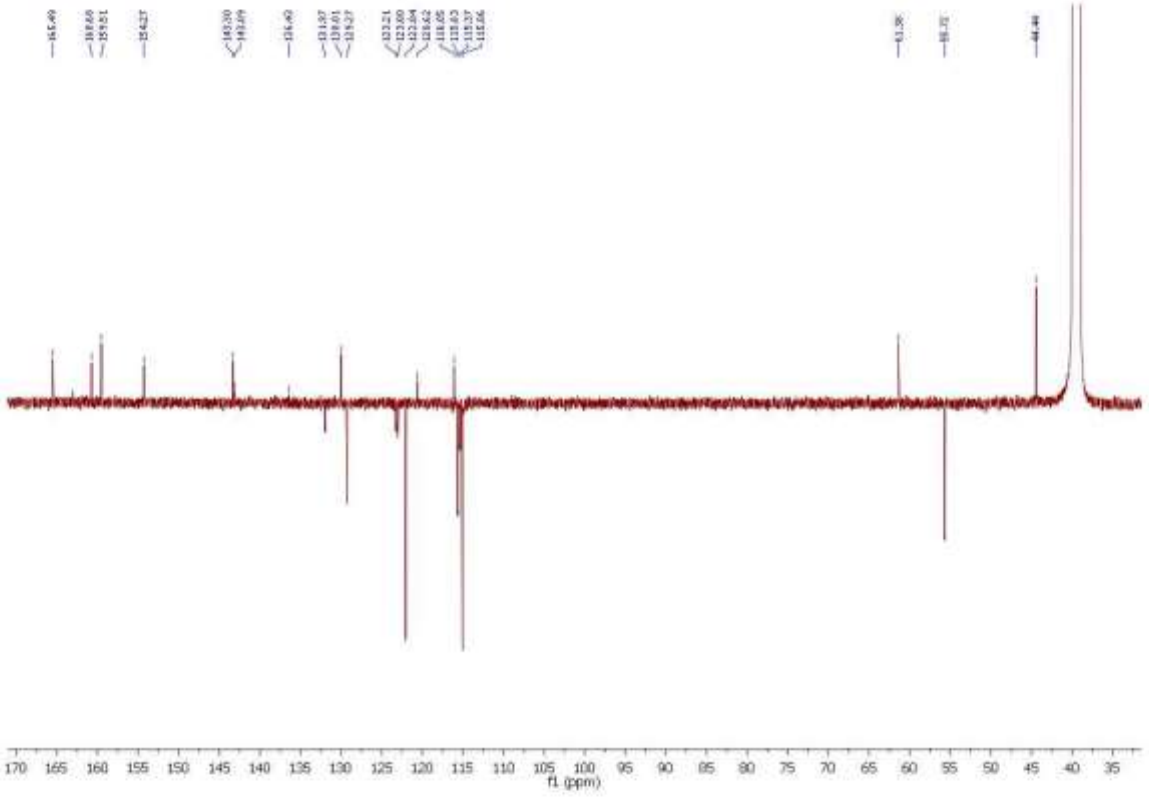

Fig. S12 a) ^{1}H NMR and b) ^{13}C NMR of compd. **9c**

a)

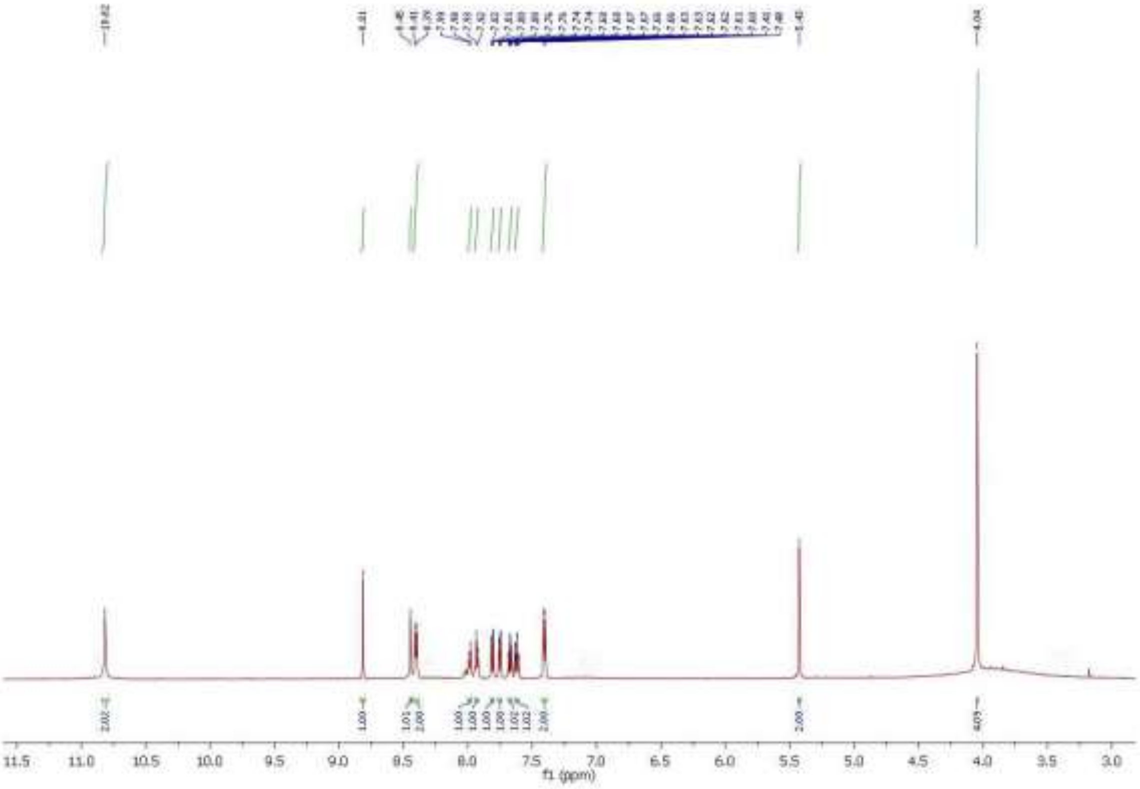

b)

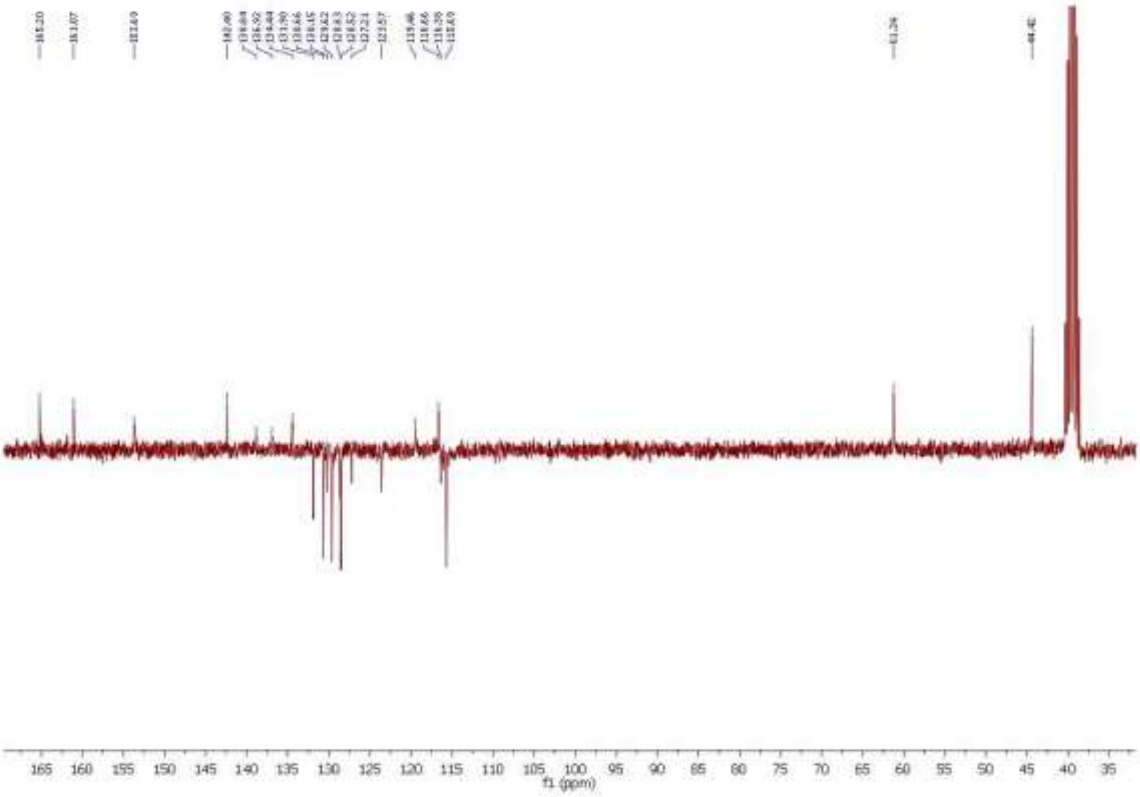

Fig. S13 a) ^{1}H NMR and b) ^{13}C NMR of compd. **9e**

a)

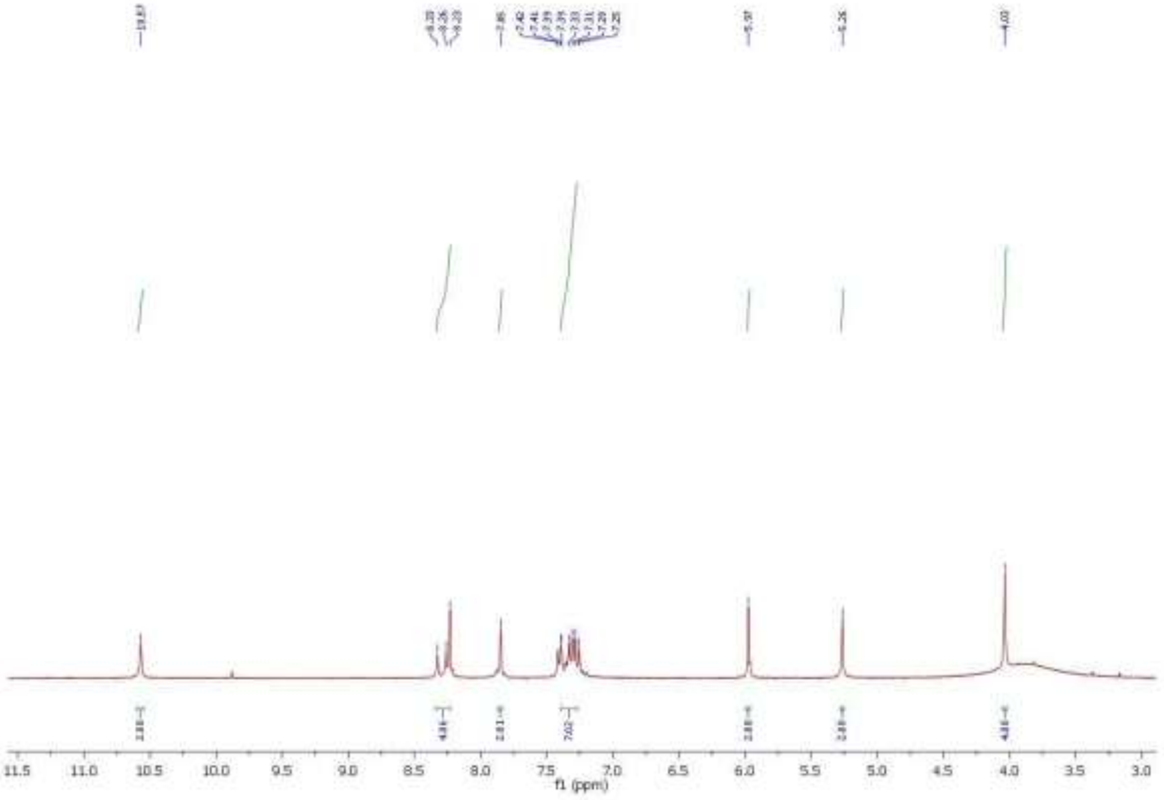

b)

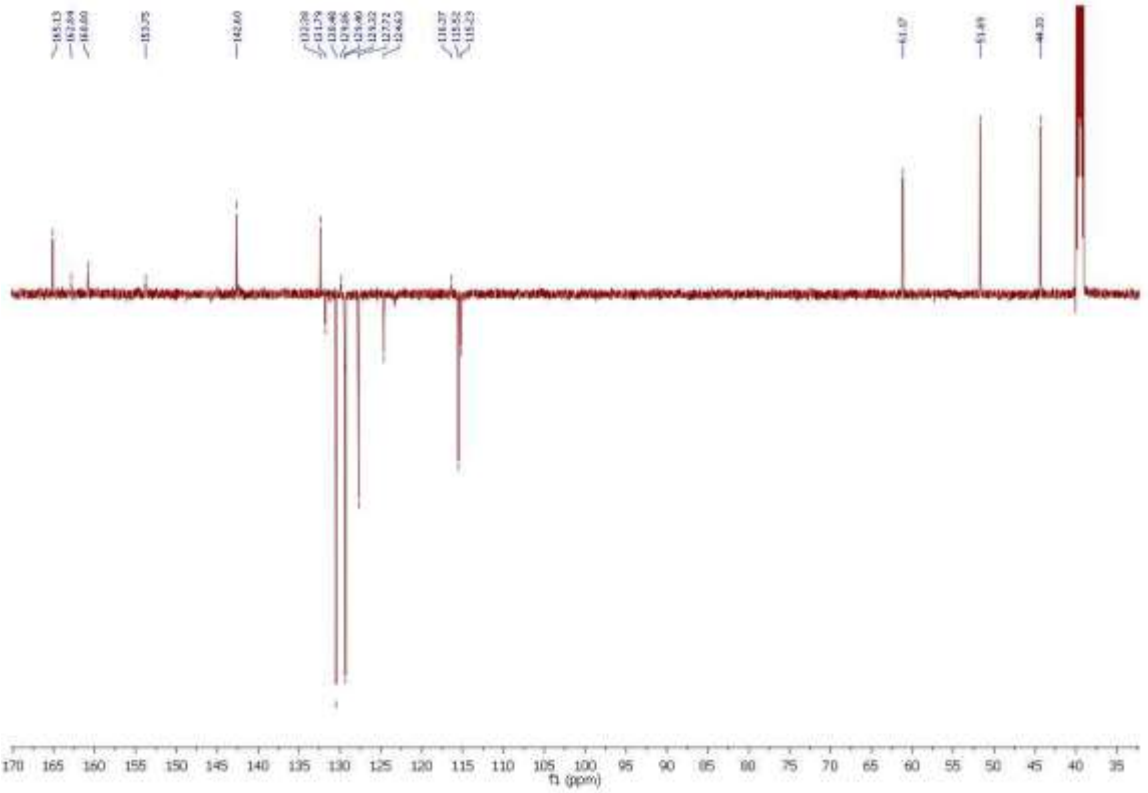

Supplement: Supplemental Material [file IENZ_A_1484733_SM4650.pdf]
